# Expression of Concern: Effect of daily physical activity on ambulatory blood pressure in pregnant women with chronic hypertension: A prospective cohort study protocol

**DOI:** 10.1371/journal.pone.0344268

**Published:** 2026-03-09

**Authors:** The PLOS One Editors

The *PLOS One* Editors issue this Expression of Concern because this article [1] was identified as one of a series of submissions for which we have concerns about the peer review process. Readers are advised to interpret the article [1] with caution.

## References

[1] Lv Y, Hu R, Liang Y, Zhou Y, Lian Y, He T. Effect of daily physical activity on ambulatory blood pressure in pregnant women with chronic hypertension: A prospective cohort study protocol. PLoS One. 2024;19(1):e0296023. doi: 10.1371/journal.pone.0296023 38198464 PMC10781089

