# Supplementary material for: Effect of daily physical activity on ambulatory blood pressure in pregnant women with chronic hypertension: A prospective cohort study protocol
Source: PLoS One. 2024 Jan 10;19(1):e0296023. doi: 10.1371/journal.pone.0296023 (PMC10781089; doi:10.1371/journal.pone.0296023)
Supplement: S1 File — (DOCX) [file pone.0296023.s001.docx]

**版本号：V1.0，版本日期：2022-04-05**

**日常体力活动对子痫前期高风险孕妇妊娠期血压的影响**

**研究方案**

1. **研究背景**

世界卫生组织研究显示子痫前期是临床上导致孕产妇死亡的第2位主要原因，占孕产妇死亡的14%^[1]^。此外，子痫前期增加了胎儿生长受限、医源性早产、母儿远期心血管疾病的风险^[2]^，严重威胁母婴的身体健康、出生人口素质及生活质量。目前为止，子痫前期的病因及发病机制尚不清楚。对于已发展为子痫前期的孕妇，临床上尚无明确有效的期待治疗措施。虽然终止妊娠是阻断子痫前期病情进展的有效手段，但是对于早发型子痫前期终止妊娠不可避免的造成了医源性早产。因此，与早诊断、早干预、早处理相比，早期排查和筛选风险因素、做好早期预警和预防对于妊娠期高血压疾病的诊治更为重要。孕妇风险因素与平均动脉压（mean arterial pressure，MAP）、胎盘生长因子（placental growth factor，PLGF）和子宫动脉搏动指数（uterine artery pulsatility index，UtA-PI）相结合（FMF算法）是目前指南推荐的孕早期预测早发型子痫前期的模型。一系列RCT和Meta分析也显示预测为子痫前期高风险的孕妇预防性使用低剂量阿司匹林可有效降低子痫前期的发病率^[3,4]^。同时，国内外妊娠期运动指南均指出运动干预可预防子痫前期的发生。那么，子痫前期高风险孕妇在预防性口服低剂量阿司匹林的基础上给予运动干预或许可进一步降低子痫前期的发生率并有利于血压控制。

1. **FMF算法是指南推荐的孕早期预测子痫前期的模型。**

Bayes（贝叶斯）定理是将母体特征和病史的风险与多种生物物理和生化检测结果相结合用于预测孕妇患子痫前期的风险。目前预测子痫前期较好的生物物理指标为MAP、PLGF和UtA-PI。英国胎儿医学基金会（FMF）的系列研究支持使用FMF算法对子痫前期的发生风险进行预测。2018年世界妇产超声指南建议在孕11-13周，联合母体因素、母体动脉血压、子宫动脉血流和PLGF作为子痫前期的筛查方案^[5]^。2019年国际妇产联盟 FIGO《孕早期子痫前期筛查和预防实用指南》中明确指出，孕妇风险因素与MAP、PLGF和UtA-PI相结合（FMF算法）是孕早期筛查早发型子痫前期最好的模型，建议所有孕妇都应进行孕早期子痫前期风险筛查^[6]^。对经FMF算法筛选为子痫前期高风险的孕妇尽早预防、干预和诊治，可降低子痫前期的发生及其导致的严重产科并发症及围产期母儿死亡。

1. **妊娠期适当运动可预防子痫前期等妊娠期并发症的发生。**

运动作为健康生活方式的重要组成部分，在生命的各个阶段都发挥着维持和提高机体心肺功能，并降低肥胖、糖尿病、高血压等慢性疾病风险的重要作用。妊娠期女性也建议保持适当的运动。妊娠期运动可通过加强机体肌肉力量缓解疼痛、减轻关节水肿，增强孕妇产程和分娩的体力，进而促进分娩、减少剖宫产。同时，妊娠期运动还可改善孕妇情绪、减少抑郁。更加重要的是，妊娠期适当运动不增加早产的发生，并可以控制孕妇妊娠期体重过度增长，减少胰岛素抵抗，预防妊娠期糖尿病、子痫前期等妊娠并发症^[7,8]^，从而改善母儿预后，保障母婴安全和健康。**国内妊娠期运动共识建议无运动禁忌证的孕妇，每周进行5 d、每次持续30 min的中等强度运动**^[9]^。多项指南均提出**妊娠期运动可预防子痫前期的发生**^[9-13]^。一项Meta分析显示，运动可使子痫前期的发生风险降低41%(OR 0.59, 95% CI 0.37 to 0.94)^[7]^。对于已诊断为子痫前期的孕妇，国内外孕期运动指南和共识虽然将子痫前期列为孕期运动的禁忌症，但同时也指出此类孕妇孕期运动的利弊还需进一步证实。运动干预是否有益于子痫前期孕妇的血压控制也需要进一步的研究。

1. **运动干预可降低高血压的发生风险，且有益于高血压患者的血压控制。**

在全球范围内，运动被推荐为控制非孕期高血压的一线辅助方法^[14.15]^。大量的研究证实，运动干预可降低高血压前期及高血压成人的血压，也可降低血压正常成人患高血压的风险。一项Meta分析^[16]^显示运动干预可使高血压前期的收缩压和舒张压均显著降低，可使血压正常的成人收缩压降低2-5mmHg，舒张压降低1-4mmHg。也有研究显示^[17]^，高强度的日常体力活动与高血压发生率降低相关。最近JAMA Cardiology发表的一项RCT研究^[18]^显示持续12周的中等强度的有氧运动可有效降低难治性高血压患者的血压。同时，越来越多的证据表明，成人高血压患者中收缩压的降低与心血管疾病发病率和死亡率的降低呈线性相关^[19]^。收缩压降低10mmHg或舒张压降低4mmHg可使脑卒中的风险降低约30%，心肌梗死风险降低20%^[20]^。因此，运动对高血压及其引起的心脑血管并发症的预防和控制均有重要的意义。

血压的管理是子痫前期孕妇诊治的一个重要部分。对于子痫前期的孕妇，由于对胎儿存在潜在影响，可选择的降压药物类型较为有限。经积极治疗高血压不可控制时，需考虑终止妊娠，以避免心脑血管意外和胎盘早剥等严重母儿并发症。目前研究证实很多非孕期高血压管理策略可以应用于妊娠期。妊娠期高血压疾病指南推荐的降压手段除药物降压，还包括生活干预，如控制食盐摄入、规律作息、戒烟戒酒、运动等。已有研究^[21]^发现运动可有益于血压正常孕妇的孕晚期血压。**运动作为治疗非孕期高血压管理的一线辅助方法，或许可使子痫前期高风险孕妇及已发生子痫前期的孕妇获益。**

**本研究拟通过FMF算法评估孕妇子痫前期的风险，并监测其妊娠期日常体力活动强度及血压变化，探讨运动强度对子痫前期高风险孕妇血压的影响，进一步为确诊子痫前期的孕妇妊娠期运动提供依据。**

**参考文献**

1. Say L, Chou D, Gemmill A, et al. Global causes of maternal death: a WHO systematic analysis. Lancet Glob Health, 2014, 2(6): e323-33.
2. Phipps EA, Thadhani R, Benzing T, et al. Pre-eclampsia: Pathogenesis, novel diagnostics and therapies. Nat Rev Nephrol, 2019, 15(5): 275-28.
3. Rolnik DL, Wright D, Poon LC, et al. Aspirin versus Placebo in Pregnancies at High Risk for Preterm Preeclampsia.The New England Journal of Medicine, 2017, 377(7): 613-622.
4. Meher S, Duley L, Hunter K, et al. Antiplatelet therapy before of after 16 weeks’ gestation for preventing preeclampsia: an individual participant data meta-analysis. Am J Obstet Gynecol, 2017, 216(2): 121-128.
5. Clinical standards committee. ISUOG Practice Guidelines: role of ultrasound in screening for and follow-up of pre-eclampsia. 2018.
6. Poon LC, Andrew S, Jonathan HA, et al. The international federation of Gynecology and obstetrics (FIGO) initiative on pre-eclampsia: A pragmatic guide for first-trimester screening and prevention. Int J Gynaecol Obstet. 2019 May; 145 Suppl 1:1-33.
7. Davenport MH, Ruchat SM, Poitras VJ, et al. Prenatal exercise for the prevention of gestational diabetes mellitus and hypertensive disorders of pregnancy: a systematic review and meta-analysis[J]. Br J Sports Med, 2018,52(21):1367-1375.
8. Wang C, Wei Y, Zhang X, et al. A randomized clinical trial of exercise during pregnancy to prevent gestational diabetes mellitus and improve pregnancy outcome in overweight and obese pregnant women[J]. Am J Obstet Gynecol, 2017, 216(4):340-351.
9. 中国妇幼保健协会妊娠合并糖尿病专业委员会,中华医学会妇产科学分会产科学组. 妊娠期运动专家共识(草案). 中华围产医学杂志,2021,24(09):641-645.
10. ACOG Committee Opinion No. 650: Physical activity and exercise during pregnancy and the postpartum period[J]. Obstet Gynecol, 2015,126(6):e135-142.
11. Mottola MF, Davenport MH, Ruchat SM, et al. 2019 Canadian guideline for physical activity throughout pregnancy[J]. Br J Sports Med, 2018, 52(21): 1339-1346.
12. Royal College of Obstetricians and Gynaecologists. Exercise in pregnancy (Statement No. 4) [EB/OL]. (2015-02-04) [2021-06-01]. https:// www. rcog. org. uk/en/guidelines-research-services/guidelines/exercise-in-pregnancy-statement-no.4/.
13. Evenson KR, Barakat R, Brown WJ, et al. Guidelines for physical activity during pregnancy: comparisons from around the world[J]. Am J Lifestyle Med, 2014,8(2):102-121.
14. Williams B, Mancia G, SpieringW, et al; ESC Scientific Document Group. 2018 ESC/ESH guidelines for the management of arterial hypertension. Eur Heart J. 2018;39(33):3021-3104.
15. Whelton PK, Carey RM, Aronow WS, et al. 2017 ACC/AHA/AAPA/ABC/ACPM/AGS/APhA/ASH/ASPC/NMA/PCNA guideline for the prevention, detection, evaluation, and management of high blood pressure in adults: a report of the American College of Cardiology/American Heart Association Task Force on Clinical Practice Guidelines. Circulation.2018;138(17): e484-e594.
16. Pescatello, LS; Buchner, DM; Jakicic, JM; et al. Physical Activity to Prevent and Treat Hypertension: A Systematic Review. Med Sci Sports Exerc.2019 06 ;51(6) :1314-1323.
17. Huai P, Xun H, Reilly KH, Wang Y, Ma W, Xi B. Physical activity and risk of hypertension: a meta-analysis of prospective cohort studies.Hypertension. 2013;62(6):1021–6.
18. Lopes, S; Mesquita-Bastos, J; Garcia, C; et al. Effect of Exercise Training on Ambulatory Blood Pressure Among Patients with Resistant Hypertension A Randomized Clinical Trial. JAMA Cardiol.2021 11 01 ;6(11) :1317-1323.
19. Bundy JD, Li C, Stuchlik P, et al. Systolic blood pressure reduction and risk of cardiovascular disease and mortality: a systematic review and network meta-analysis. JAMA Cardiol. 2017;2(7):775-781.
20. Staessen JA, Wang JG, Thijs L. Cardiovascular protection and blood pressure reduction: ameta-analysis. Lancet. 2001;358(9290):1305-1315.
21. Sobierajski, FM; Purdy, GM; Usselman, CW; et al. Maternal Physical Activity Is Associated with Improved Blood Pressure Regulation During Late Pregnancy. Can J Cardiol.2018 04 ;34(4) :485-491
22. **研究方案**
23. **研究类型：**前瞻性队列研究
24. **研究对象**
25. **纳入标准**
26. ≥18周岁；
27. 妊娠11-13^+6^周；
28. 利用FMF算法（母体因素+MAP+PLGF+UtA-PI）计算子痫前期高风险的孕妇；
29. 单胎妊娠、胎儿存活；
30. 同意参加并签署知情同意书。
31. **排除标准(在纳入标准内排除不符合条件的患者)**
32. 严重心脏或呼吸系统疾病；
33. 甲状腺功能亢进；
34. 糖尿病；
35. 宫颈机能不全；
36. 既往复发性流产史
37. 既往非子痫前期引起的早产史；
38. 此次妊娠先兆流产、稽留流产；
39. 胎盘前置状态；
40. 重度贫血、营养不良或极低体重（体重指数＜12kg/m^2^）；
41. 胎儿严重畸形或异常（无胎心搏动）；
42. 严重精神障碍，无法表达意愿者；
43. 存在明显其他异常体征、实验室检查或其他临床疾病，经研究者判断，不适合参加研究者；
44. 无法获得随访及分娩信息者。
45. **诊断标准（2020年中国妊娠期高血压疾病诊治指南）**

**3.1子痫前期：**妊娠20周后孕妇出现收缩压≥140mmHg和（或）舒张压≥90mmHg，伴有下列任意1项：尿蛋白定量≥0.3g/24h，或尿蛋白/肌酐比值≥0.3，或随机尿蛋白≥（+）（无条件进行蛋白定量时的检查方法）；无蛋白尿但伴有以下任何1种器官或系统受累：心、肺、肝、肾等重要器官，或血液系统、消化系统、神经系统的异常改变，胎盘-胎儿受到累及等。

**3.2重度子痫前期**（severe pre-eclampsia）为子痫前期孕妇出现下述任一表现者：

- - 1. 血压持续升高不可控制：收缩压≥160mmHg和（或）舒张压≥110mmHg；
    2. 持续性头痛、视觉障碍或其他中枢神经系统异常表现；
    3. 持续性上腹部疼痛及肝包膜下血肿或肝破裂表现；
    4. 转氨酶水平异常：血丙氨酸转氨酶（ALT）或天冬氨酸转氨酶（AST）水平升高；
    5. 肾功能受损：尿蛋白定量＞2.0g/24h；少尿（24h尿量＜400ml，或每小时尿量＜17ml），或血肌酐水平＞106μmol/L；
    6. 低蛋白血症伴腹水、胸水或心包积液；
    7. 血液系统异常：血小板计数呈持续性下降并低于100×10^9^/L；微血管内溶血，表现有贫血、血乳酸脱氢酶（LDH）水平升高或黄疸；
    8. 心功能衰竭；
    9. 肺水肿；
    10. 胎儿生长受限或羊水过少、胎死宫内、胎盘早剥等。

1. **终点事件：**
2. 终止妊娠
3. 出现以下情况：阴道出血、规律并有痛觉的宫缩、胎膜早破、呼吸困难、头晕、头痛、胸痛、肌肉无力影响平衡
4. 孕期出现其他严重临床疾病，经研究者判断，不适合继续进行研究者。
5. **分组方案**
6. 分组方法：早孕（11~13^+6^周）手腕佩戴ActiGraph wGT3X-BT运动加速度计，监测1周内日常体力活动强度、时间、步数及能量代谢，同时收集手机APP运动步数。按照早孕期活动强度和活动时间分为轻度、中度活动两组。中等活动强度且时间达每周150min为中度活动组，轻度活动强度或活动时间未达到每周150min为轻度活动组。
7. 药物干预方案：两组孕妇均根据指南推荐妊娠11~13^+6^周开始每天服用小剂量阿司匹林100mg，直至终止妊娠前2-7天或妊娠34周；口服钙补充量至少为1 g/d。
8. 健康指导：控制食盐摄入（<6 g/d）、戒烟、规律作息、控制体重等。
9. **运动监测：**手腕佩戴ActiGraph wGT3X-BT运动加速度计，监测一周的体力活动。除洗澡、游泳等接触水时取下，其余时间需按要求佩戴，确保每天佩戴10 h以上，且至少工作日（ 周一至周五）有2 天、休息日（周六至周日）有1 天的有效数据。
10. **活动强度：**分为轻度体力活动(100–1952 CPM),中度以上活动(≥1952 CPM)，结束后通过ActiLife 6.0分析软件进行数据采集。
11. **活动代谢率：**以平均代谢当量（metabolic equivalents，METs）来评价。
12. **自觉劳累分级：**使用基于Borg感知运动强度度量表（表1）的自觉劳累分级（ratings of perceived exertion, RPE）。Borg量表有从6~20分共15个等级评分，代表对劳累程度感受的不同等级，其中6分代表“非常非常轻松”，20分代表“非常非常困难”。对于中等强度的运动，孕妇的RPE评分应为13~14分，即其对自我运动强度的感受为有点困难。


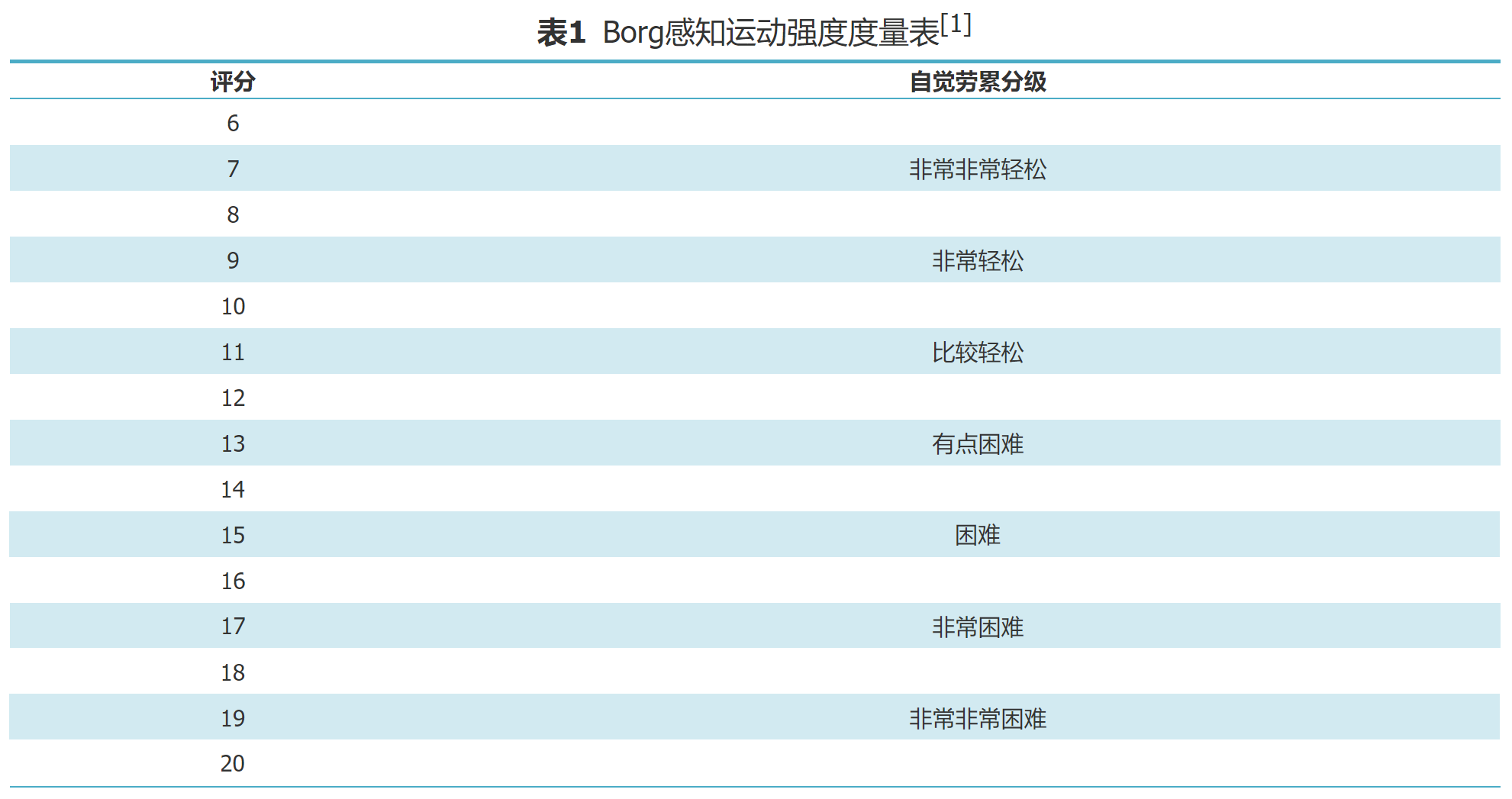


1. **结局指标**
2. 主要结局指标：孕中、晚期24小时动态血压收缩压相对基线变化的差值；
3. 次要结局指标：孕中、晚期动态血压白天及夜间收缩压、白天夜间及24小时舒张压、诊室血压相对基线变化的差值；
4. 探索性指标：子痫前期的发生率；运动对子痫前期孕妇控制血压的作用
5. 安全性指标：活动耐量下降率；胸闷气短发生率；早期心衰或急性左心衰的发生率；
6. 其它相关性指标：子痫前期严重并发症（子痫、心功能衰竭、 肺水肿、 HELLP综合征、 DIC）及胎死宫内、医源性早产、新生儿窒息、NICU入住率。口服阿司匹林、钙剂的依从性；手机运动记录监测运动强度依从性；主观用力程度量表（RPE）监测个体的运动强度耐受性。
7. **研究流程**

**
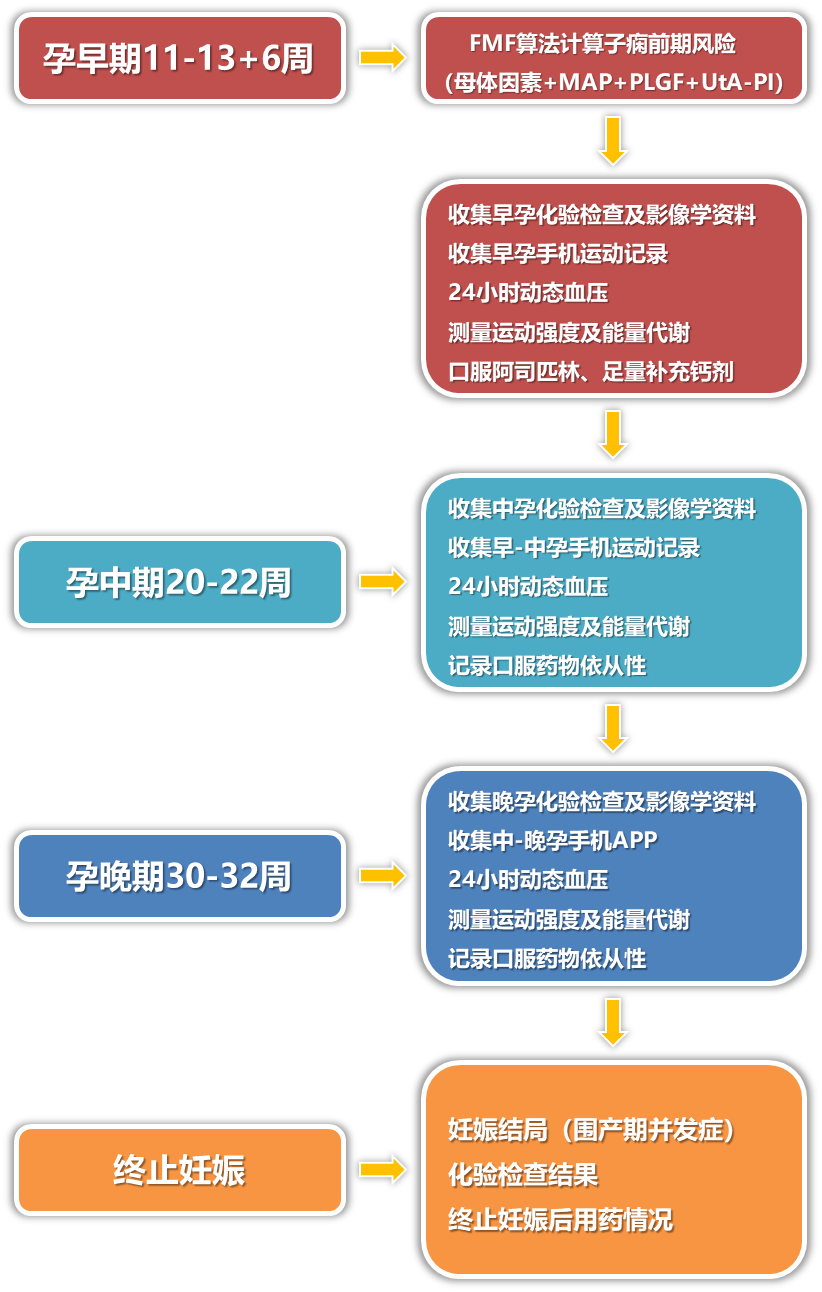
**

1. **信息采集时点及内容**
2. **入组登记资料（11-13^+6^）**
   1. **孕妇人口学特征及病史（危险因素）**
3. 年龄、种族、身高、体重、孕周（CRL计算方式）、职业；
4. 怀孕方式（自然妊娠、 辅助生殖技术受孕--使用排卵药或体外受精）；
5. 吸烟史；
6. 慢性高血压史；
7. I型或II型糖尿病史；
8. 肾脏疾病史；
9. 异常妊娠史（21/18-三体、NTD、不明流产史、妊娠期糖尿病等）；
10. 子痫前期史；
11. 子痫前期家族史（母亲或姐妹）；
12. 自身免疫病史（系统性红斑狼疮或抗磷脂酶综合征）；
13. 既往孕史及胎次；
14. 妊娠间隔时间是否超过10年；
15. 收缩压≥130mmHg或舒张压≥80mmHg；
16. 存在高血压危险因素如阻塞性睡眠呼吸暂停；
17. 既往产前诊断经历（绒毛活检/羊水穿刺）。
    1. **实验室检查**

包括叶酸、维生素B12、空腹血糖、血常规、尿常规（尿蛋白、尿糖、尿酮体、尿潜血、尿白细胞）、肾功能（血清肌酐、血尿素氮、血尿酸、尿蛋白肌酐比）、血脂、凝血功能、肝功能、甲状腺功能。

- 1. **体格检查**

孕11-13^+6^周双臂血压、BMI（身高、体重）。

- 1. **超声检查**

孕11-13^+6^周子宫动脉搏动指数、头臀长、NT。

- 1. **血清标志物检查**

孕11-13^+6^周胎盘生长因子（PLGF）。

- 1. **主要结局指标的基线值**

24小时动态血压、早孕手机APP运动记录、手腕佩戴ActiGraph wGT3X-BT运动加速度计监测运动强度及能量代谢。

1. **随访资料登记（中、晚孕）**
   1. **主要结局指标**

24小时动态血压、早孕手机运动记录、手腕佩戴ActiGraph wGT3X-BT运动加速度计监测运动强度及能量代谢

- 1. **子痫前期的表现**

出现血压升高收缩压≥140 mmHg和（或）舒张压≥90 mmHg；伴有下列任意1项：尿蛋白定量≥0.3g/24h，或尿蛋白/肌酐比值≥0.3，或随机尿蛋白≥（+）；或无尿蛋白但伴有以下任何1种器官或系统受累

- 1. 中枢神经系统异常表现：头痛、头晕视觉障碍或其他；
  2. 持续性上腹部疼痛及肝包膜下血肿或肝破裂表现；
  3. 转氨酶水平异常：血丙氨酸转氨酶（ALT）或天冬氨酸转氨酶（AST）水平升高；
  4. 肾功能受损：少尿（24 h尿量<400 ml，或每小时尿量<17 ml），或血肌酐水平>106 μmol/L；
  5. 低蛋白血症伴腹水、胸水或心包积液；
  6. 血液系统异常：血小板计数呈持续性下降并低于100×10^9^/L；微血管内溶血，表现有贫血、血乳酸脱氢酶（LDH）水平升高或黄疸；
  7. 心功能衰竭；
  8. 肺水肿；
  9. 胎儿生长受限或羊水过少、胎死宫内、胎盘早剥。
  10. **实验室检查**

空腹血糖、血常规、尿常规（尿蛋白、尿糖、尿酮体、尿潜血、尿白细胞）、肾功能（血清肌酐、血尿素氮、血尿酸、尿蛋白肌酐比）、血脂、凝血功能、肝功能、甲状腺功能。

- 1. **体格检查**

双臂血压、身高、体重。

- 1. **超声检查**

头臀长、双顶径、脐动脉搏动指数、脐动脉阻力指数、脐动脉S/D比值。

- 1. **血清标志物筛查**

孕26-27^+6^周及32-36^+6^周血清胎盘生长因子(PLGF)｡

- 1. **用药依从性**

每次随访携带医嘱服用的药物，对药物服用数量进行统计。

- 1. **其他检查**

宫高、腹围。

1. **数据管理及统计分析**

7.1 数据管理

本研究拟采用电子采集和管理系统，研究人员负责原始资料的收集，并负责组织将病例报告信息录入电子表格中。数据管理过程中所发现的问题将采用质询表（query form）的形式通知研究者，由研究者进行审核，并将对质询的答复尽快反馈，反馈记录作为数据修改的依据保存，病例审核完成后，对数据进行锁定，锁定的数据文件不允许再做变动。

7.2 统计分析

7.2.1数据质量评估及数据清理

研究要求评估数据的完整性、准确性、一致性是否满足预先设定的质量要求，对重复的数据和不合格的数据进行清理，形成可用的数据。

7.2.2 数据分析

样本量估算：本研究的主要结局指标为孕中、晚期两组间动态血压白天收缩压与基线相比变化的差值。依据主要结局指标计算样本量，设α=0.05（双侧），β=0.10，两组的样本量采用1:1的比例。Susana Lopes等报道，难治性高血压患者经过为期12周的中等强度有氧运动训练，试验组和对照组白天收缩压较基线变化差值分别为-7.3 ± 12.7mmHg、1.1 ± 8.2mmHg，利用PASS11软件，分别计算得到两组的样本量分别为N1=N2=36例。假定研究对象的失访率为20%，则需样本量N1=N2=36÷0.8≈45例。实际研究中，两组各纳入45例，共90例。

采用SPSS 25.0统计软件分析数据。Shapiro-Wilk检验确定数据分布的正态性。正态性分布的计量资料用$\overline{x}$±s表示。偏态分布的计量资料用中位数M、四分位区间（P25，P75）表示。计数资料用计数资料以百分率表示。Wilcoxon和Fisher精确检验用于评估组间基线特征的差异。主要结局指标和次要结局指标为孕中、晚期血压相对基线的变化值，采用重复测量方差分析。采用意向性治疗分析（ITT分析）、并与符合方案集分析比较，根据是否合并慢性高血压进行亚组分析。P＜0.05为差异有统计学意义。

1. **研究质量控制**

研究过程中，定期监查，以保证研究方案的所有内容都得到严格遵守和研究资料的填写的正确、规范。

1）研究开始前，进行研究人员的培训，统一记录方式和判断标准。培训内容包括但不限于：研究方案、研究涉及的SOP、研究问卷及表格、知情同意、伦理、不良事件报告、相关疾病知识和医学知识。

2）研究者应按要求如实、详细、认真记录研究表格中的各项内容，以确保内容真实、可靠。

3）临床研究中所有观察结果都应加以核实，以保证数据的可靠，确保临床研究中各项结论均有对应的原始记录。在临床研究和数据处理阶段均有相应的数据管理措施。

4）定期抽样检查病例报告表，并核查原始文件。

5）为保证参与者的安全性和保障数据准确、完整并可靠，研究者应当保留原始病例观察表、临床病历和患者的来诊记录等作为本研究的原始记录。如有要求，研究者应当能够提供这些原始记录文件。

1. **知情同意、伦理审查及法规问题**

9.1知情同意

研究人员需确保入组孕妇充分了解本项目的研究性质、研究内容、检查内容、研究时限及数据归属、可能发生的风险及获益、隐私保护等，并获得已签署姓名及日期的书面知情同意。确保患者有机会询问并有时间考虑知情同意的内容，确保及时回答患者在研究过程中的任何问题。

9.2 伦理审查

在开始临床研究之前，研究方案终稿、书面知情同意书最终版本及其他提供给患者的相关书面信息必须得到伦理委员会的批准和书面同意。研究者必须确保把这些文件递交给相应的伦理委员会和研究机构人员。伦理委员会的观点必须以书面形式表示。

9.3 法律法规

本研究的实施符合《世界医学学会赫尔辛基宣言》，并符合我国现行的《体外诊断试剂临床研究技术指导原则》及诊断试剂临床试验相关法律法规的伦理原则。

1. **资料保存**

为保证国家相关单位和责任单位对临床研究的评价与监督，研究者应同意保存所有研究相关资料，包括对患者的确认（能有效地核对不同的记录资料，如病例报告表和原始病例）、原始的有签名的知情同意书、研究表格的详细记录等。保存期为临床研究结束后3年。

1. **保密性及数据发表**

本项目的研究方案、研究者手册、受试者信息及在研究数据发表前本研究生成的任何研究数据均属保密信息，不得泄露。本研究产生的数据归研究单位及申办者所有，研究单位有权发表或出版本研究相关数据。本项目的研究人员享有在出版、发表的研究著作中署名的权利。

**版本号：V2.0，版本日期：2022-07-05**

**日常体力活动对慢性高血压孕妇妊娠期血压的影响**

**研究方案**

1. **研究背景**

世界卫生组织研究显示子痫前期是临床上导致孕产妇死亡的第2位主要原因，占孕产妇死亡的14%^[1]^。此外，妊娠期高血压疾病增加了胎儿生长受限、医源性早产、母儿远期心血管疾病的风险^[2]^，严重威胁母婴的身体健康、出生人口素质及生活质量。目前为止，其病因及发病机制尚不清楚。对于已发展为子痫前期的孕妇，临床上尚无明确有效的期待治疗措施。虽然终止妊娠是阻断子痫前期病情进展的有效手段，但是对于早发型子痫前期终止妊娠不可避免的造成了医源性早产。国内外妊娠期运动指南均指出运动干预可预防子痫前期的发生。那么，慢性高血压孕妇的运动干预或许可有利于预防子痫前期的发生和血压控制。

1. **慢性高血压孕妇是子痫前期及相关并发症的高风险人群。**

慢性高血压影响了0.3%至4.3%的孕妇，慢性高血压孕妇发生子痫前期的风险是血压正常孕妇的5.43倍。此外，慢性高血压与剖宫产、孕产妇死亡率、早产、死产、小胎龄(SGA)、低出生体重和新生儿重症监护病房住院的高风险相关[3,4]。与单独的子痫前期相比，慢性高血压合并子痫前期更容易发生上述孕产妇及围产期不良结局[5,6]。在患有慢性高血压的孕妇的围产期，血压管理是非常重要的。两项大型随机试验[7,8]发现，针对慢性高血压患者140/90 mmHg的降压治疗对孕产妇和新生儿是安全且有益的。然而，由于可能或已证实的胎儿毒性问题，某些药物在妊娠期应避免使用[9-11]。与非妊娠期相比，孕妇抗高血压药物的选择有限。当高血压经积极治疗无法控制时，应考虑终止妊娠，以避免胎盘早剥等母婴并发症和心脑血管不良事件的发生。生活方式的改变可以增强降压治疗的效果。妊娠期和非妊娠期高血压指南[12-14]建议改变生活方式，包括减少盐、戒烟和戒酒、健康饮食和饮酒、减轻体重和定期体育锻炼。

1. **妊娠期适当运动可预防妊娠期并发症的发生。**

运动作为健康生活方式的重要组成部分，在生命的各个阶段都发挥着维持和提高机体心肺功能，并降低肥胖、糖尿病、高血压等慢性疾病风险的重要作用。妊娠期女性也建议保持适当的运动。妊娠期运动可通过加强机体肌肉力量缓解疼痛、减轻关节水肿，增强孕妇产程和分娩的体力，进而促进分娩、减少剖宫产。同时，妊娠期运动还可改善孕妇情绪、减少抑郁。更加重要的是，妊娠期适当运动不增加早产的发生，并可以控制孕妇妊娠期体重过度增长，减少胰岛素抵抗，预防妊娠期糖尿病、子痫前期等妊娠并发症[15,16]，从而改善母儿预后，保障母婴安全和健康。**国内妊娠期运动共识建议无运动禁忌证的孕妇，每周进行5 d、每次持续30 min的中等强度运动**。多项指南均提出**妊娠期运动可预防子痫前期的发生**[17-21]。一项Meta分析显示，运动可使子痫前期的发生风险降低41%(OR 0.59, 95% CI 0.37 to 0.94)^[15]^。对于已诊断为子痫前期的孕妇，国内外孕期运动指南和共识虽然将子痫前期列为孕期运动的禁忌症，但同时也指出此类孕妇孕期运动的利弊还需进一步证实。运动干预是否有益于子痫前期孕妇的血压控制也需要进一步的研究。

1. **运动干预可降低高血压的发生风险，且有益于高血压患者的血压控制。**

在全球范围内，运动被推荐为控制非孕期高血压的一线辅助方法^[22.23]^。大量的研究证实，运动干预可降低高血压前期及高血压成人的血压，也可降低血压正常成人患高血压的风险。一项Meta分析[24]显示运动干预可使高血压前期的收缩压和舒张压均显著降低，可使血压正常的成人收缩压降低2-5mmHg，舒张压降低1-4mmHg。也有研究显示[25]，高强度的日常体力活动与高血压发生率降低相关。最近JAMA Cardiology发表的一项RCT研究[26]显示持续12周的中等强度的有氧运动可有效降低难治性高血压患者的血压。同时，越来越多的证据表明，成人高血压患者中收缩压的降低与心血管疾病发病率和死亡率的降低呈线性相关[27]。收缩压降低10mmHg或舒张压降低4mmHg可使脑卒中的风险降低约30%，心肌梗死风险降低20%[28]。因此，运动对高血压及其引起的心脑血管并发症的预防和控制均有重要的意义。

血压的管理是慢性高血压孕妇诊治的一个重要部分。由于对胎儿存在潜在影响，慢性高血压孕妇可选择的降压药物类型较为有限。经积极治疗高血压不可控制时，需考虑终止妊娠，以避免心脑血管意外和胎盘早剥等严重母儿并发症。运动作为治疗非孕期高血压管理的一线辅助方法，或许可使慢性高血压孕妇获益。已有研究[29]发现运动可有益于血压正常孕妇的孕晚期血压。一项观察性队列研究表明，坚持有益的生活方式可能会显著降低妊娠高血压疾病(HDP)后慢性高血压的风险，但没有明确的证据表明体育锻炼可以改变HDP与慢性高血压之间的关系。两项研究[30,31]对116名患有慢性高血压和/或先兆子痫的孕妇进行了研究，结果表明，每周一次使用固定自行车进行体育锻炼对分娩方式、孕产妇和新生儿发病率、血压和心率的变化没有影响。现有的研究很少，不足以指导慢性高血压孕妇的妊娠护理。有必要开展研究，探索并帮助解决有关慢性高血压妇女怀孕期间体育锻炼的问题，如益处和危害，多久一次，多远，多长时间，以何种强度。

**本研究拟通过监测其慢性高血压孕妇妊娠期日常体力活动强度及血压变化，探讨运动强度对慢性高血压孕妇血压的影响，进一步为慢性高血压孕妇妊娠期运动提供依据。**

**参考文献**

1. Say L, Chou D, Gemmill A, et al. Global causes of maternal death: a WHO systematic analysis. Lancet Glob Health, 2014, 2(6): e323-33.
2. Phipps EA, Thadhani R, Benzing T, et al. Pre-eclampsia: Pathogenesis, novel diagnostics and therapies. Nat Rev Nephrol, 2019, 15(5): 275-28.
3. Al Khalaf SY, O'Reilly EJ, Barrett PM, DF BL, Pawley LC, McCarthy FP, et al. Impact of Chronic Hypertension and Antihypertensive Treatment on Adverse Perinatal Outcomes: Systematic Review and Meta-Analysis. J Am Heart Assoc. 2021;10(9):e018494.
4. Bramham K, Parnell B, Nelson-Piercy C, Seed PT, Poston L, Chappell LC. Chronic hypertension and pregnancy outcomes: systematic review and meta-analysis. BMJ. 2014;348:g2301.
5. Valent AM, DeFranco EA, Allison A, Salem A, Klarquist L, Gonzales K, et al. Expectant management of mild preeclampsia versus superimposed preeclampsia up to 37 weeks. Am J Obstet Gynecol. 2015;212(4):515 e1-8.
6. Rezk M, Gamal A, Emara M. Maternal and fetal outcome in de novo preeclampsia in comparison to superimposed preeclampsia: a two-year observational study. Hypertens Pregnancy. 2015;34(2):137-44.
7. Magee LA, von Dadelszen P, Rey E, Ross S, Asztalos E, Murphy KE, et al. Less-tight versus tight control of hypertension in pregnancy. N Engl J Med. 2015;372(5):407-17.
8. Tita AT, Szychowski JM, Boggess K, Dugoff L, Sibai B, Lawrence K, et al. Treatment for Mild Chronic Hypertension during Pregnancy. N Engl J Med. 2022;386(19):1781-92.
9. Bellos I, Pergialiotis V, Papapanagiotou A, Loutradis D, Daskalakis G. Comparative efficacy and safety of oral antihypertensive agents in pregnant women with chronic hypertension: a network metaanalysis. Am J Obstet Gynecol. 2020;223(4):525-37.
10. Ahmed B, Tran DT, Zoega H, Kennedy SE, Jorm LR, Havard A. Maternal and perinatal outcomes associated with the use of renin-angiotensin system (RAS) blockers for chronic hypertension in early pregnancy. Pregnancy Hypertens. 2018;14:156-61.
11. Bateman BT, Patorno E, Desai RJ, Seely EW, Mogun H, Dejene SZ, et al. Angiotensin-Converting Enzyme Inhibitors and the Risk of Congenital Malformations. Obstet Gynecol. 2017;129(1):174-84.
12. Hypertensive Disorders in Pregnancy Subgroup CSoO, Gynecology CMA. [Diagnosis and treatment of hypertension and pre-eclampsia in pregnancy: a clinical practice guideline in China2020]. Zhonghua Fu Chan Ke Za Zhi. 2020;55(4):227-38.
13. ACOG Practice Bulletin No. 202: Gestational Hypertension and Preeclampsia. Obstet Gynecol. 2019;133(1):1.
14. Unger T, Borghi C, Charchar F, Khan NA, Poulter NR, Prabhakaran D, et al. 2020 International Society of Hypertension Global Hypertension Practice Guidelines. Hypertension. 2020;75(6):1334-57.
15. Davenport MH, Ruchat SM, Poitras VJ, et al. Prenatal exercise for the prevention of gestational diabetes mellitus and hypertensive disorders of pregnancy: a systematic review and meta-analysis[J]. Br J Sports Med, 2018,52(21):1367-1375.
16. Wang C, Wei Y, Zhang X, et al. A randomized clinical trial of exercise during pregnancy to prevent gestational diabetes mellitus and improve pregnancy outcome in overweight and obese pregnant women[J]. Am J Obstet Gynecol, 2017, 216(4):340-351.
17. 中国妇幼保健协会妊娠合并糖尿病专业委员会,中华医学会妇产科学分会产科学组. 妊娠期运动专家共识(草案). 中华围产医学杂志,2021,24(09):641-645.
18. ACOG Committee Opinion No. 650: Physical activity and exercise during pregnancy and the postpartum period[J]. Obstet Gynecol, 2015,126(6):e135-142.
19. Mottola MF, Davenport MH, Ruchat SM, et al. 2019 Canadian guideline for physical activity throughout pregnancy[J]. Br J Sports Med, 2018, 52(21): 1339-1346.
20. Royal College of Obstetricians and Gynaecologists. Exercise in pregnancy (Statement No. 4) [EB/OL]. (2015-02-04) [2021-06-01]. https:// www. rcog. org. uk/en/guidelines-research-services/guidelines/exercise-in-pregnancy-statement-no.4/.
21. Evenson KR, Barakat R, Brown WJ, et al. Guidelines for physical activity during pregnancy: comparisons from around the world[J]. Am J Lifestyle Med, 2014,8(2):102-121.
22. Williams B, Mancia G, SpieringW, et al; ESC Scientific Document Group. 2018 ESC/ESH guidelines for the management of arterial hypertension. Eur Heart J. 2018;39(33):3021-3104.
23. Whelton PK, Carey RM, Aronow WS, et al. 2017 ACC/AHA/AAPA/ABC/ACPM/AGS/APhA/ASH/ASPC/NMA/PCNA guideline for the prevention, detection, evaluation, and management of high blood pressure in adults: a report of the American College of Cardiology/American Heart Association Task Force on Clinical Practice Guidelines. Circulation.2018;138(17): e484-e594.
24. Pescatello, LS; Buchner, DM; Jakicic, JM; et al. Physical Activity to Prevent and Treat Hypertension: A Systematic Review. Med Sci Sports Exerc.2019 06 ;51(6) :1314-1323.
25. Huai P, Xun H, Reilly KH, Wang Y, Ma W, Xi B. Physical activity and risk of hypertension: a meta-analysis of prospective cohort studies.Hypertension. 2013;62(6):1021–6.
26. Lopes, S; Mesquita-Bastos, J; Garcia, C; et al. Effect of Exercise Training on Ambulatory Blood Pressure Among Patients with Resistant Hypertension A Randomized Clinical Trial. JAMA Cardiol.2021 11 01 ;6(11) :1317-1323.
27. Bundy JD, Li C, Stuchlik P, et al. Systolic blood pressure reduction and risk of cardiovascular disease and mortality: a systematic review and network meta-analysis. JAMA Cardiol. 2017;2(7):775-781.
28. Staessen JA, Wang JG, Thijs L. Cardiovascular protection and blood pressure reduction: ameta-analysis. Lancet. 2001;358(9290):1305-1315.
29. Sobierajski, FM; Purdy, GM; Usselman, CW; et al. Maternal Physical Activity Is Associated with Improved Blood Pressure Regulation During Late Pregnancy. Can J Cardiol.2018 04 ;34(4) :485-491
30. Kasawara KT, Burgos CS, do Nascimento SL, Ferreira NO, Surita FG, Pinto ESJL. Maternal and Perinatal Outcomes of Exercise in Pregnant Women with Chronic Hypertension and/or Previous Preeclampsia: A Randomized Controlled Trial. ISRN Obstet Gynecol. 2013;2013:857047.
31. Burgos CS, Kasawara KT, Costa ML, Pinto ESJL. PP041. The effect of exercise in pregnant women with chronic hypertension and/or previous preeclampsia on blood pressure and heart rate variability. Pregnancy Hypertens. 2012;2(3):263-4.
32. **研究方案**
33. **研究类型：**前瞻性队列研究
34. **研究对象**
35. **纳入标准**
36. ≥18周岁；
37. 妊娠11-13^+6^周；
38. 慢性高血压孕妇；
39. 单胎妊娠、胎儿存活；
40. 同意参加并签署知情同意书。
41. **排除标准(在纳入标准内排除不符合条件的患者)**
42. 严重心脏或呼吸系统疾病；
43. 甲状腺功能亢进；
44. 糖尿病；
45. 宫颈机能不全；
46. 既往复发性流产史
47. 既往非子痫前期引起的早产史；
48. 此次妊娠先兆流产、稽留流产；
49. 胎盘前置状态；
50. 重度贫血、营养不良或极低体重（体重指数＜12kg/m^2^）；
51. 胎儿严重畸形或异常（无胎心搏动）；
52. 严重精神障碍，无法表达意愿者；
53. 存在明显其他异常体征、实验室检查或其他临床疾病，经研究者判断，不适合参加研究者；
54. 无法获得随访及分娩信息者。
55. **诊断标准（2020年中国妊娠期高血压疾病诊治指南）**

**3.1子痫前期：**妊娠20周后孕妇出现收缩压≥140mmHg和（或）舒张压≥90mmHg，伴有下列任意1项：尿蛋白定量≥0.3g/24h，或尿蛋白/肌酐比值≥0.3，或随机尿蛋白≥（+）（无条件进行蛋白定量时的检查方法）；无蛋白尿但伴有以下任何1种器官或系统受累：心、肺、肝、肾等重要器官，或血液系统、消化系统、神经系统的异常改变，胎盘-胎儿受到累及等。

**3.2重度子痫前期**（severe pre-eclampsia）为子痫前期孕妇出现下述任一表现者：

- - 1. 血压持续升高不可控制：收缩压≥160mmHg和（或）舒张压≥110mmHg；
    2. 持续性头痛、视觉障碍或其他中枢神经系统异常表现；
    3. 持续性上腹部疼痛及肝包膜下血肿或肝破裂表现；
    4. 转氨酶水平异常：血丙氨酸转氨酶（ALT）或天冬氨酸转氨酶（AST）水平升高；
    5. 肾功能受损：尿蛋白定量＞2.0g/24h；少尿（24h尿量＜400ml，或每小时尿量＜17ml），或血肌酐水平＞106μmol/L；
    6. 低蛋白血症伴腹水、胸水或心包积液；
    7. 血液系统异常：血小板计数呈持续性下降并低于100×10^9^/L；微血管内溶血，表现有贫血、血乳酸脱氢酶（LDH）水平升高或黄疸；
    8. 心功能衰竭；
    9. 肺水肿；
    10. 胎儿生长受限或羊水过少、胎死宫内、胎盘早剥等。

1. **终点事件：**
2. 终止妊娠
3. 出现以下情况：阴道出血、规律并有痛觉的宫缩、胎膜早破、呼吸困难、头晕、头痛、胸痛、肌肉无力影响平衡
4. 孕期出现其他严重临床疾病，经研究者判断，不适合继续进行研究者。
5. **分组方案**
6. 分组方法：早孕（11~13^+6^周）手腕佩戴ActiGraph wGT3X-BT运动加速度计，监测1周内日常体力活动强度、时间、步数及能量代谢，同时收集手机APP运动步数。按照早孕期活动强度和活动时间分为轻度、中度活动两组。中等活动强度且时间达每周150min为中度活动组，轻度活动强度或活动时间未达到每周150min为轻度活动组。
7. 药物干预方案：两组孕妇均根据指南推荐妊娠11~13^+6^周开始每天服用小剂量阿司匹林100mg，直至终止妊娠前2-7天或妊娠34周；口服钙补充量至少为1 g/d。
8. 健康指导：控制食盐摄入（<6 g/d）、戒烟、规律作息、控制体重等。
9. **运动监测：**手腕佩戴ActiGraph wGT3X-BT运动加速度计，监测一周的体力活动。除洗澡、游泳等接触水时取下，其余时间需按要求佩戴，确保每天佩戴10 h以上，且至少工作日（ 周一至周五）有2 天、休息日（周六至周日）有1 天的有效数据。
10. **活动强度：**分为轻度体力活动(100–1952 CPM),中度以上活动(≥1952 CPM)，结束后通过ActiLife 6.0分析软件进行数据采集。
11. **活动代谢率：**以平均代谢当量（metabolic equivalents，METs）来评价。
12. **自觉劳累分级：**使用基于Borg感知运动强度度量表（表1）的自觉劳累分级（ratings of perceived exertion, RPE）。Borg量表有从6~20分共15个等级评分，代表对劳累程度感受的不同等级，其中6分代表“非常非常轻松”，20分代表“非常非常困难”。对于中等强度的运动，孕妇的RPE评分应为13~14分，即其对自我运动强度的感受为有点困难。


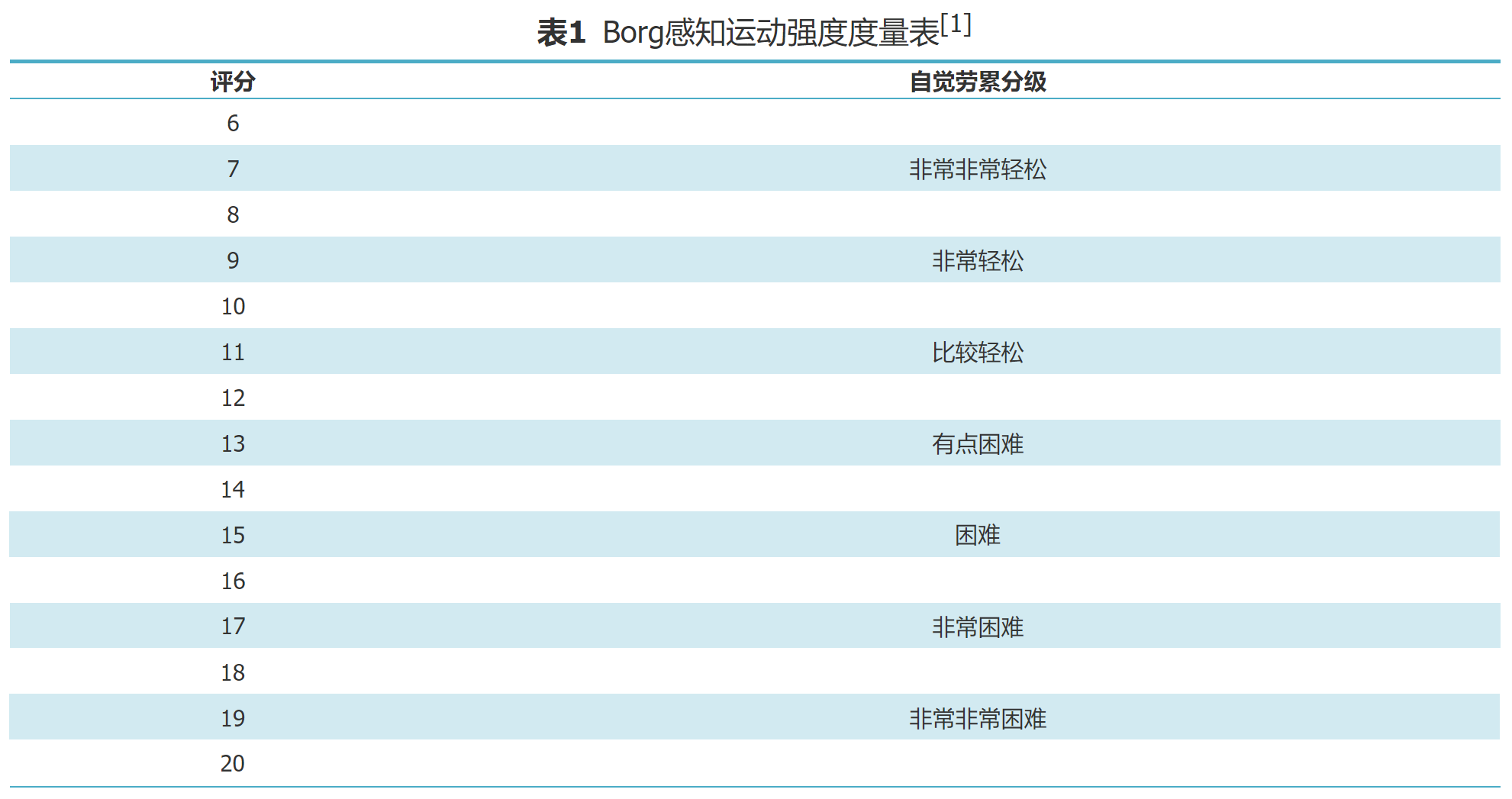


1. **结局指标**
2. 主要结局指标：孕晚期24小时动态血压收缩压相对基线变化的差值；
3. 次要结局指标：孕中期24小时动态血压收缩压相对基线变化的差值；孕中、晚期动态血压白天及夜间收缩压、白天夜间及24小时舒张压、诊室血压相对基线变化的差值；
4. 探索性指标：子痫前期的发生率；运动对子痫前期孕妇控制血压的作用
5. 安全性指标：活动耐量下降率；胸闷气短发生率；早期心衰或急性左心衰的发生率；
6. 其它相关性指标：子痫前期严重并发症（子痫、心功能衰竭、 肺水肿、 HELLP综合征、 DIC）及胎死宫内、医源性早产、新生儿窒息、NICU入住率。口服阿司匹林、钙剂的依从性；手机运动记录监测运动强度依从性；主观用力程度量表（RPE）监测个体的运动强度耐受性。
7. **研究流程**

**
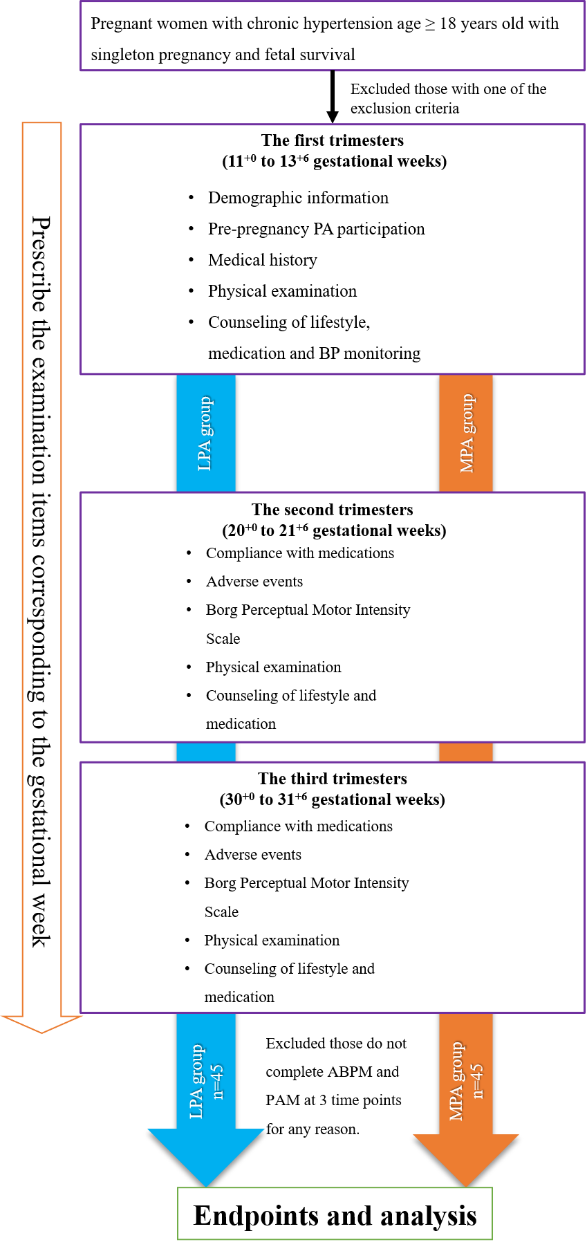
**

1. **信息采集时点及内容**
2. **入组登记资料（11-13^+6^）**
   1. **孕妇人口学特征及病史（危险因素）**
3. 年龄、种族、身高、体重、孕周（CRL计算方式）、职业；
4. 怀孕方式（自然妊娠、 辅助生殖技术受孕--使用排卵药或体外受精）；
5. 吸烟史；
6. 慢性高血压史；
7. I型或II型糖尿病史；
8. 肾脏疾病史；
9. 异常妊娠史（21/18-三体、NTD、不明流产史、妊娠期糖尿病等）；
10. 子痫前期史；
11. 子痫前期家族史（母亲或姐妹）；
12. 自身免疫病史（系统性红斑狼疮或抗磷脂酶综合征）；
13. 既往孕史及胎次；
14. 妊娠间隔时间是否超过10年；
15. 收缩压≥130mmHg或舒张压≥80mmHg；
16. 存在高血压危险因素如阻塞性睡眠呼吸暂停；
17. 既往产前诊断经历（绒毛活检/羊水穿刺）。
    1. **实验室检查**

包括叶酸、维生素B12、空腹血糖、血常规、尿常规（尿蛋白、尿糖、尿酮体、尿潜血、尿白细胞）、肾功能（血清肌酐、血尿素氮、血尿酸、尿蛋白肌酐比）、血脂、凝血功能、肝功能、甲状腺功能。

- 1. **体格检查**

孕11-13^+6^周双臂血压、BMI（身高、体重）。

- 1. **超声检查**

孕11-13^+6^周子宫动脉搏动指数、头臀长、NT。

- 1. **血清标志物检查**

孕11-13^+6^周胎盘生长因子（PLGF）。

- 1. **主要结局指标的基线值**

24小时动态血压、早孕手机APP运动记录、手腕佩戴ActiGraph wGT3X-BT运动加速度计监测运动强度及能量代谢。

1. **随访资料登记（中、晚孕）**
   1. **主要结局指标**

24小时动态血压、早孕手机运动记录、手腕佩戴ActiGraph wGT3X-BT运动加速度计监测运动强度及能量代谢

- 1. **子痫前期的表现**

出现血压升高收缩压≥140 mmHg和（或）舒张压≥90 mmHg；伴有下列任意1项：尿蛋白定量≥0.3g/24h，或尿蛋白/肌酐比值≥0.3，或随机尿蛋白≥（+）；或无尿蛋白但伴有以下任何1种器官或系统受累

- 1. 中枢神经系统异常表现：头痛、头晕视觉障碍或其他；
  2. 持续性上腹部疼痛及肝包膜下血肿或肝破裂表现；
  3. 转氨酶水平异常：血丙氨酸转氨酶（ALT）或天冬氨酸转氨酶（AST）水平升高；
  4. 肾功能受损：少尿（24 h尿量<400 ml，或每小时尿量<17 ml），或血肌酐水平>106 μmol/L；
  5. 低蛋白血症伴腹水、胸水或心包积液；
  6. 血液系统异常：血小板计数呈持续性下降并低于100×10^9^/L；微血管内溶血，表现有贫血、血乳酸脱氢酶（LDH）水平升高或黄疸；
  7. 心功能衰竭；
  8. 肺水肿；
  9. 胎儿生长受限或羊水过少、胎死宫内、胎盘早剥。
  10. **实验室检查**

空腹血糖、血常规、尿常规（尿蛋白、尿糖、尿酮体、尿潜血、尿白细胞）、肾功能（血清肌酐、血尿素氮、血尿酸、尿蛋白肌酐比）、血脂、凝血功能、肝功能、甲状腺功能。

- 1. **体格检查**

双臂血压、身高、体重。

- 1. **超声检查**

头臀长、双顶径、脐动脉搏动指数、脐动脉阻力指数、脐动脉S/D比值。

- 1. **血清标志物筛查**

孕26-27^+6^周及32-36^+6^周血清胎盘生长因子(PLGF)｡

- 1. **用药依从性**

每次随访携带医嘱服用的药物，对药物服用数量进行统计。

- 1. **其他检查**

宫高、腹围。

1. **数据管理及统计分析**

7.1 数据管理

本研究拟采用电子采集和管理系统，研究人员负责原始资料的收集，并负责组织将病例报告信息录入电子表格中。数据管理过程中所发现的问题将采用质询表（query form）的形式通知研究者，由研究者进行审核，并将对质询的答复尽快反馈，反馈记录作为数据修改的依据保存，病例审核完成后，对数据进行锁定，锁定的数据文件不允许再做变动。

7.2 统计分析

7.2.1数据质量评估及数据清理

研究要求评估数据的完整性、准确性、一致性是否满足预先设定的质量要求，对重复的数据和不合格的数据进行清理，形成可用的数据。

7.2.2 数据分析

样本量估算：本研究的主要结局指标为孕晚期两组间动态血压白天收缩压与基线相比变化的差值。依据主要结局指标计算样本量，设α=0.05（双侧），β=0.10，两组的样本量采用1:1的比例。Susana Lopes等报道，难治性高血压患者经过为期12周的中等强度有氧运动训练，试验组和对照组白天收缩压较基线变化差值分别为-7.3 ± 12.7mmHg、1.1 ± 8.2mmHg，利用PASS11软件，分别计算得到两组的样本量分别为N1=N2=36例。假定研究对象的失访率为20%，则需样本量N1=N2=36÷0.8≈45例。实际研究中，两组各纳入45例，共90例。

采用SPSS 25.0统计软件分析数据。Shapiro-Wilk检验确定数据分布的正态性。正态性分布的计量资料用$\overline{x}$±s表示。偏态分布的计量资料用中位数M、四分位区间（P25，P75）表示。计数资料用计数资料以百分率表示。Wilcoxon和Fisher精确检验用于评估组间基线特征的差异。主要结局指标和次要结局指标为孕中、晚期血压相对基线的变化值，采用重复测量方差分析。采用意向性治疗分析（ITT分析）、并与符合方案集分析比较，根据是否合并慢性高血压进行亚组分析。P＜0.05为差异有统计学意义。

1. **研究质量控制**

研究过程中，定期监查，以保证研究方案的所有内容都得到严格遵守和研究资料的填写的正确、规范。

1）研究开始前，进行研究人员的培训，统一记录方式和判断标准。培训内容包括但不限于：研究方案、研究涉及的SOP、研究问卷及表格、知情同意、伦理、不良事件报告、相关疾病知识和医学知识。

2）研究者应按要求如实、详细、认真记录研究表格中的各项内容，以确保内容真实、可靠。

3）临床研究中所有观察结果都应加以核实，以保证数据的可靠，确保临床研究中各项结论均有对应的原始记录。在临床研究和数据处理阶段均有相应的数据管理措施。

4）定期抽样检查病例报告表，并核查原始文件。

5）为保证参与者的安全性和保障数据准确、完整并可靠，研究者应当保留原始病例观察表、临床病历和患者的来诊记录等作为本研究的原始记录。如有要求，研究者应当能够提供这些原始记录文件。

1. **知情同意、伦理审查及法规问题**

9.1知情同意

研究人员需确保入组孕妇充分了解本项目的研究性质、研究内容、检查内容、研究时限及数据归属、可能发生的风险及获益、隐私保护等，并获得已签署姓名及日期的书面知情同意。确保患者有机会询问并有时间考虑知情同意的内容，确保及时回答患者在研究过程中的任何问题。

9.2 伦理审查

在开始临床研究之前，研究方案终稿、书面知情同意书最终版本及其他提供给患者的相关书面信息必须得到伦理委员会的批准和书面同意。研究者必须确保把这些文件递交给相应的伦理委员会和研究机构人员。伦理委员会的观点必须以书面形式表示。

9.3 法律法规

本研究的实施符合《世界医学学会赫尔辛基宣言》，并符合我国现行的《体外诊断试剂临床研究技术指导原则》及诊断试剂临床试验相关法律法规的伦理原则。

1. **资料保存**

为保证国家相关单位和责任单位对临床研究的评价与监督，研究者应同意保存所有研究相关资料，包括对患者的确认（能有效地核对不同的记录资料，如病例报告表和原始病例）、原始的有签名的知情同意书、研究表格的详细记录等。保存期为临床研究结束后3年。

1. **保密性及数据发表**

本项目的研究方案、研究者手册、受试者信息及在研究数据发表前本研究生成的任何研究数据均属保密信息，不得泄露。本研究产生的数据归研究单位及申办者所有，研究单位有权发表或出版本研究相关数据。本项目的研究人员享有在出版、发表的研究著作中署名的权利。
