# Supplementary material for: Effect of daily physical activity on ambulatory blood pressure in pregnant women with chronic hypertension: A prospective cohort study protocol
Source: PLoS One. 2024 Jan 10;19(1):e0296023. doi: 10.1371/journal.pone.0296023 (PMC10781089; doi:10.1371/journal.pone.0296023)
Supplement: S2 File — (DOCX) [file pone.0296023.s002.docx]

Version number: V1.0, date: 2022-04-05

**Effect of Physical Activity in Leisure Time on Ambulatory Blood Pressure Among Pregnant Women at High Risk for Preeclampsia**

**Background**

Pre-eclampsia, which is the second leading cause of maternal death [1], increases the risk of fetal growth restriction, iatrogenic premature delivery, and long-term cardiovascular diseases [2], and threatens the health of the mother and child. The etiology of preeclampsia remains unclear. There is no effective method for expectant treatment except termination of pregnancy which inevitably leads to iatrogenic premature delivery. Therefore, early prediction and prevention are important. The prediction model which combines the risk factors with mean arterial pressure (MAP), placental growth factor (PLGF) and uterine artery pulsatility index (UtA-PI) is recommended by guidelines to predict early-onset preeclampsia. A series of RCTs and meta-analyses have shown that prophylactic use of low-dose aspirin in pregnant women effectively reduces the incidence of preeclampsia [3,4]. Guidelines involving exercise during pregnancy indicate that exercise intervention reduces the occurrence of preeclampsia. Exercise in addition to prophylactic low-dose aspirin in pregnant women at high risk of preeclampsia may further reduce the incidence of preeclampsia and benefit blood pressure control.

Bayes' theorem combines maternal characteristics and medical history with biophysical and biochemical tests to predict the risk of preeclampsia in pregnant women. The biophysical indicators of predictive value include MAP, PLGF and UtA-PI. A series of studies recommend predicting the risk of preeclampsia by FMF algorithms. Guidelines of obstetrics and gynecology recommend a combination of maternal factors, arterial blood pressure, uterine arterial flow, and PLGF as screening protocols for preeclampsia at 11-13 weeks of pregnancy [5]. In the guideline for screening and prevention of preeclampsia, it is recommended that all pregnant women should undergo risk screening for early-onset preeclampsia [6]. Early prevention, intervention, diagnosis and treatment of pregnant women at high risk of preeclampsia screened by FMF algorithm reduce the incidence of preeclampsia and severe complications.

Exercise plays an important role in maintaining and improving cardiopulmonary function and reducing the risk of obesity, diabetes, hypertension and other chronic diseases at all stages of life. Pregnant women are also advised to maintain moderate exercise. Pregnancy exercise reduces joint edema, enhances physical strength during delivery, relieves pain, and reduces the incidence of cesarean section. Exercise during pregnancy also reduces depression. Moderate exercise during pregnancy does not increase premature birth and is helpful to control weight gain during pregnancy, reduce insulin resistance, and prevent gestational diabetes, preeclampsia and other pregnancy complications [7,8]. The consensus on exercise during pregnancy suggests that pregnant women without exercise contraindications should carry out moderate-intensity exercise lasting 30 minutes for 5 days every week [9]. Multiple guidelines have proposed that exercise during pregnancy could reduce the incidence of preeclampsia [9-13]. A meta-analysis showed that exercise reduced the risk of preeclampsia by 41% (OR 0.59, 95% CI 0.37 to 0.94) [7]. For pregnant women diagnosed with preeclampsia, preeclampsia is a contraindication for pregnancy exercise. However, the advantages and disadvantages of pregnancy exercise for such women need to be further confirmed. Further research is also needed to determine whether exercise interventions benefit blood pressure control in women with preeclampsia.

Globally, exercise is recommended as a first-line adjunct to the control of non-gestational hypertension [14.15]. A large number of studies have confirmed that exercise intervention can reduce blood pressure in adults with prehypertension and hypertension, and also reduce the risk of hypertension in adults with normal blood pressure. A meta-analysis [16] showed that exercise intervention can significantly reduce both systolic and diastolic blood pressure in prehypertension, and reduce systolic blood pressure by 2-5mmHg and diastolic blood pressure by 1-4mmHg in adults with normal blood pressure. Studies [17] have also shown that high-intensity daily physical activity is associated with a lower incidence of hypertension. A recent RCT study [18] published in JAMA Cardiology showed that 12 weeks of moderate-intensity aerobic exercise was effective in reducing blood pressure in patients with refractory hypertension. At the same time, more and more evidence has shown that reduced systolic blood pressure in adults with hypertension is linearly correlated with the reduction of cardiovascular disease morbidity and mortality [19]. A reduction of 10mmHg in systolic blood pressure or 4mmHg in diastolic blood pressure can reduce the risk of stroke by about 30% and myocardial infarction by 20% [20]. Therefore, exercise plays an important role in the prevention and control of hypertension and its cardiovascular and cerebrovascular complications.

Blood pressure management is an important part of the diagnosis and treatment of pregnant women with preeclampsia. For pregnant women with preeclampsia, the options for medications are limited due to potential effects on the fetus. Termination of pregnancy should be considered when hypertension is uncontrollable after treatment to avoid serious maternal and fetal complications such as cardiovascular and cerebrovascular accidents and placental abruption. In addition to drugs, life interventions are recommended in the guidelines for hypertensive diseases during pregnancy, such as controlling salt intake, regular rest, quitting smoking and drinking, and exercise. Previous studies [21] have found that exercise can benefit the blood pressure of pregnant women with normal blood pressure in late pregnancy. As a first-line adjunct to the management of non-pregnant hypertension, exercise may benefit pregnant women at high risk of preeclampsia and those with preeclampsia.

This study aims to evaluate the risk of preeclampsia in pregnant women by using FMF algorithm, and monitor the changes in the intensity of daily physical activity and blood pressure during pregnancy, explore the influence of exercise intensity on the blood pressure of pregnant women at high risk of preeclampsia, and further provide evidence for the diagnosis of exercise during pregnancy in pregnant women with preeclampsia.

**Reference**

1. Say L, Chou D, Gemmill A, et al. Global causes of maternal death: a WHO systematic analysis. Lancet Glob Health, 2014, 2(6): e323-33.
2. Phipps EA, Thadhani R, Benzing T, et al. Pre-eclampsia: Pathogenesis, novel diagnostics and therapies. Nat Rev Nephrol, 2019, 15(5): 275-28.
3. Rolnik DL, Wright D, Poon LC, et al. Aspirin versus Placebo in Pregnancies at High Risk for Preterm Preeclampsia.The New England Journal of Medicine, 2017, 377(7): 613-622.
4. Meher S, Duley L, Hunter K, et al. Antiplatelet therapy before of after 16 weeks’ gestation for preventing preeclampsia: an individual participant data meta-analysis. Am J Obstet Gynecol, 2017, 216(2): 121-128.
5. Clinical standards committee. ISUOG Practice Guidelines: role of ultrasound in screening for and follow-up of pre-eclampsia. 2018.
6. Poon LC, Andrew S, Jonathan HA, et al. The international federation of Gynecology and obstetrics (FIGO) initiative on pre-eclampsia: A pragmatic guide for first-trimester screening and prevention. Int J Gynaecol Obstet. 2019 May; 145 Suppl 1:1-33.
7. Davenport MH, Ruchat SM, Poitras VJ, et al. Prenatal exercise for the prevention of gestational diabetes mellitus and hypertensive disorders of pregnancy: a systematic review and meta-analysis[J]. Br J Sports Med, 2018,52(21):1367-1375.
8. Wang C, Wei Y, Zhang X, et al. A randomized clinical trial of exercise during pregnancy to prevent gestational diabetes mellitus and improve pregnancy outcome in overweight and obese pregnant women[J]. Am J Obstet Gynecol, 2017, 216(4):340-351.
9. 中国妇幼保健协会妊娠合并糖尿病专业委员会,中华医学会妇产科学分会产科学组. 妊娠期运动专家共识(草案). 中华围产医学杂志,2021,24(09):641-645.
10. ACOG Committee Opinion No. 650: Physical activity and exercise during pregnancy and the postpartum period[J]. Obstet Gynecol, 2015,126(6):e135-142.
11. Mottola MF, Davenport MH, Ruchat SM, et al. 2019 Canadian guideline for physical activity throughout pregnancy[J]. Br J Sports Med, 2018, 52(21): 1339-1346.
12. Royal College of Obstetricians and Gynaecologists. Exercise in pregnancy (Statement No. 4) [EB/OL]. (2015-02-04) [2021-06-01]. https:// www. rcog. org. uk/en/guidelines-research-services/guidelines/exercise-in-pregnancy-statement-no.4/.
13. Evenson KR, Barakat R, Brown WJ, et al. Guidelines for physical activity during pregnancy: comparisons from around the world[J]. Am J Lifestyle Med, 2014,8(2):102-121.
14. Williams B, Mancia G, SpieringW, et al; ESC Scientific Document Group. 2018 ESC/ESH guidelines for the management of arterial hypertension. Eur Heart J. 2018;39(33):3021-3104.
15. Whelton PK, Carey RM, Aronow WS, et al. 2017 ACC/AHA/AAPA/ABC/ACPM/AGS/APhA/ASH/ASPC/NMA/PCNA guideline for the prevention, detection, evaluation, and management of high blood pressure in adults: a report of the American College of Cardiology/American Heart Association Task Force on Clinical Practice Guidelines. Circulation.2018;138(17): e484-e594.
16. Pescatello, LS; Buchner, DM; Jakicic, JM; et al. Physical Activity to Prevent and Treat Hypertension: A Systematic Review. Med Sci Sports Exerc.2019 06 ;51(6) :1314-1323.
17. Huai P, Xun H, Reilly KH, Wang Y, Ma W, Xi B. Physical activity and risk of hypertension: a meta-analysis of prospective cohort studies.Hypertension. 2013;62(6):1021–6.
18. Lopes, S; Mesquita-Bastos, J; Garcia, C; et al. Effect of Exercise Training on Ambulatory Blood Pressure Among Patients with Resistant Hypertension A Randomized Clinical Trial. JAMA Cardiol.2021 11 01 ;6(11) :1317-1323.
19. Bundy JD, Li C, Stuchlik P, et al. Systolic blood pressure reduction and risk of cardiovascular disease and mortality: a systematic review and network meta-analysis. JAMA Cardiol. 2017;2(7):775-781.
20. Staessen JA, Wang JG, Thijs L. Cardiovascular protection and blood pressure reduction: ameta-analysis. Lancet. 2001;358(9290):1305-1315.
21. Sobierajski, FM; Purdy, GM; Usselman, CW; et al. Maternal Physical Activity Is Associated with Improved Blood Pressure Regulation During Late Pregnancy. Can J Cardiol.2018 04 ;34(4) :485-491

**Method**

**Inclusion criteria**

1. Aged ≥18 years;

2. 11-13+6 weeks of gestation;

3. Pregnant women at high risk for preeclampsia;

4. Single pregnancy and fetal survival;

5. Understand and sign the consent form.

**Exclusion criteria**

1. Severe cardiovascular or respiratory disease;

2. Hyperthyroidism;

3. Diabetes mellitus;

4. Incompetent cervix;

5. Recurrent spontaneous abortion.

6. A history of spontaneous preterm birth.

7. Threatened abortion or missed abortion

8. Placenta Praevia

9. Severe anemia, malnutrition, or very low body weight (body mass index<12kg/m2)

10. There is no fetal heartbeat during the 11-13 weeks of pregnancy screening

11. With severe mental disorders and disabled to express their will

12. With other obvious abnormal signs, laboratory examination and other clinical diseases, researchers think were not suitable for this research.

13. Loss of follow up

**Diagnostic criteria** (2020 Chinese Guidelines for the Diagnosis and Treatment of Hypertensive Diseases during Pregnancy)

**Preeclampsia**

Systolic blood pressure ≥140mmHg and/or diastolic blood pressure ≥90mmHg after 20 weeks of gestation, accompanied by any of the following: urinary protein quantity ≥0.3g/24h, or urinary protein/creatinine ratio ≥0.3, or urinary protein ≥ (+); or accompanied by following damages of organs involved: heart, lung, liver, kidney and other important organs, or abnormal in the digestive system, nervous system, placenta - fetal involvement, etc.

**Severe pre-eclampsia refers to a pregnant woman with one of the following symptoms:**

1) Uncontrolled blood pressure: systolic blood pressure ≥160mmHg and/or diastolic blood pressure ≥110mmHg;

2) Persistent headache, visual disturbance, or other central nervous system abnormalities;

3) Persistent upper abdominal pain, subcapsular hematoma or liver rupture;

4) Elevated serum alanine aminotransferase (ALT) or aspartate aminotransferase (AST) levels;

5) Impaired renal function: urinary protein content > 2.0g/24h; oliguria (24h urine volume < 400ml, or < 17ml/hour), or serum creatinine level > 106μmol/L;

6) Hypoproteinemia with ascites, pleural effusion or pericardial effusion;

7) Platelet count decreased and lower than 100×10^9^/L; hemolysis, anemia, elevated lactate dehydrogenase (LDH), or jaundice;

8) Heart failure;

9) Pulmonary edema;

10) Fetal growth restriction or oligohydramnios, fetal death, placental abruption, etc.

**End events**

1) Termination of pregnancy

2) The following conditions occur: vaginal bleeding, regular and painful contractions, premature rupture of membranes, dyspnea, dizziness, headache, chest pain, muscle weakness affecting balance

3) Other serious clinical diseases which are not suitable for further study.

**Grouping scheme**

1. Grouping method: During early pregnancy (11~13+6 weeks), an ActiGraph wGT3X-BT exercise accelerometer will be worn on the wrist to monitor the intensity, time, number of steps and energy metabolism of daily physical activity within 1 week, and the number of exercise steps will be collected by mobile phone APP. Participants will be divided into light and moderate groups according to the activity intensity of early pregnancy. Moderate-intensity activity is defined as 150 minutes per week, and the light-intensity activity group has a activity intensity or activity time of fewer than 150 minutes per week.

2. Drug intervention plan: pregnant women in both groups take low-dose aspirin 100mg daily from 11 to 13+6 gestational weeks until 2 to 7 days before termination or 34 gestational weeks as recommended by the guidelines. Oral calcium supplementation should be at least 1 g/ day.

3. Health guidance: limit salt intake (<6 g/d), smoking cessation, regular rest, weight control, etc.

4. Exercise monitoring: Wear an ActiGraph wGT3X-BT exercise accelerometer on wrist to monitor physical activity for a week. Except for bathing, swimming and other contacts with water, the rest of the time should be worn as required, ensure that more than 10 hours of wear every day, and at least two working days (Monday to Friday), rest day (Saturday to Sunday) with one day of valid data.

1) Activity intensity: light physical activity (100 -- 1952 CPM) and moderate physical activity (≥1952 CPM). Data were collected by ActiLife 6.0 analysis software after completion.

2) Active metabolic rate: measured by average metabolic equivalents (METs).

3) Perceived exertion rating: ratings of perceived exertion (RPE) based on the Borg perceived Exertion Scale (Table 1) are used. The Borg scale has 15 ratings ranging from 6 to 20, representing different levels of fatigue, with 6 being "very, very easy" and 20 beings "very, very difficult". For moderate-intensity exercise, the RPE score of pregnant women should be 13 to 14 points, that is, the feeling of self-exercise intensity is somewhat difficult.


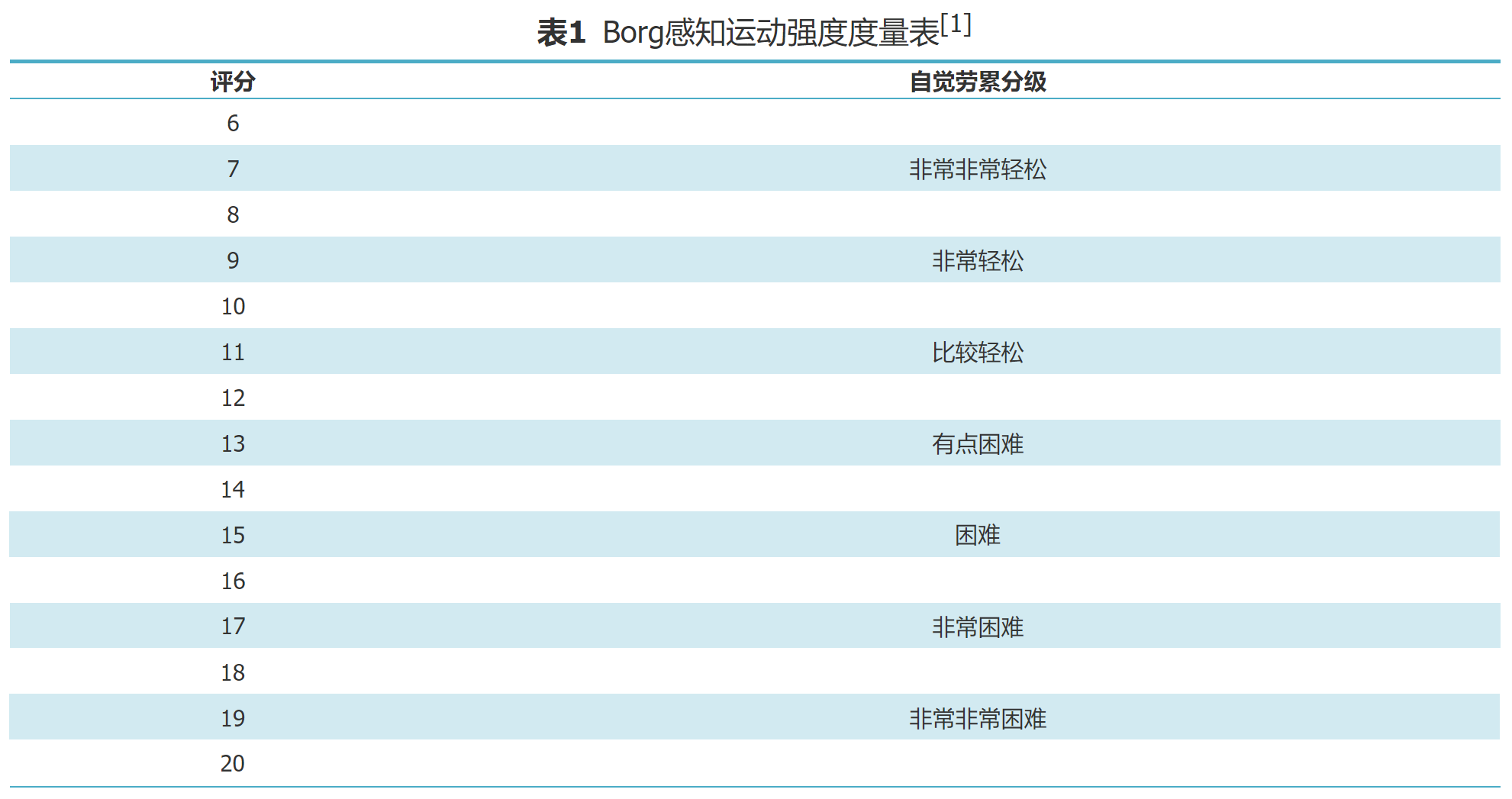


**Outcomes**

1. Primary outcome: the difference in 24-hour ambulatory systolic blood pressure change from baseline during the second and third trimesters of pregnancy;

2. Secondary outcomes: the difference of daytime and nighttime ambulatory blood pressure, daytime and nighttime and 24-hour diastolic blood pressure, and clinic blood pressure changes from baseline;

3. Exploratory outcomes: incidence of preeclampsia; effect of exercise on blood pressure control in pregnant women with preeclampsia

4. Safety outcomes: activity tolerance decline rate; incidence of shortness of breath; incidence of heart failure;

5. Other relevant outcomes: severe complications of preeclampsia (eclampsia, heart failure, pulmonary edema, HELLP syndrome, DIC), fetal death, iatrogenic premature delivery, neonatal asphyxia, NICU. Compliance with oral aspirin and calcium; mobile phone exercise recording monitoring exercise intensity compliance; the Subjective Exertion Scale (RPE) monitors an individual's exercise intensity tolerance.


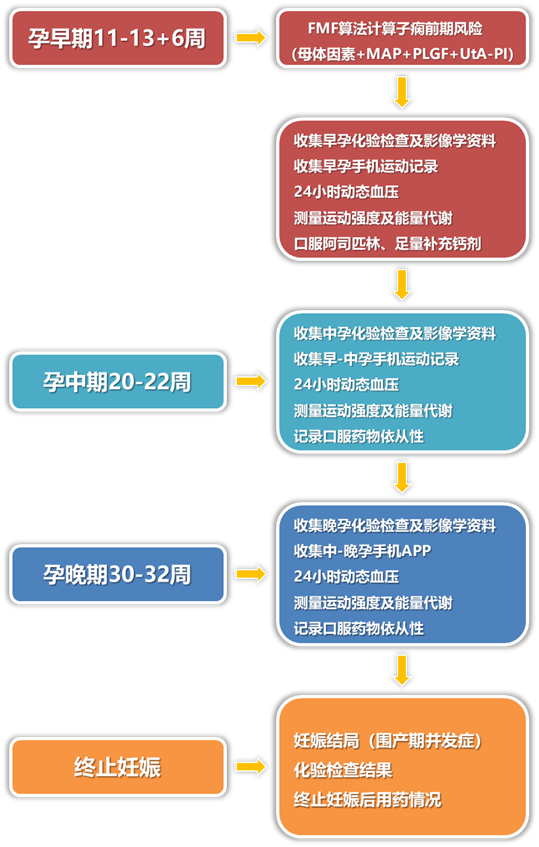
**Procedure**

**Time and content of information collection**

1 Registration Information (11-13+6)

1.1 Demographic characteristics and medical history of pregnant women (risk factors)

1) Age, race, height, weight, gestational age (CRL calculation method), occupation;

2) Method of pregnancy (natural pregnancy, assisted reproductive technology - using ovulation drugs or in vitro fertilization);

3) Smoking history;

4) History of chronic hypertension;

5) History of type I or type II diabetes;

6) History of kidney disease;

7) Abnormal pregnancy history (21/18-trisomy, NTD, unknown abortion history, gestational diabetes, etc.);

8) History of preeclampsia;

9) Family history of preeclampsia (mother or sister);

10) History of autoimmune disease (systemic lupus erythematosus or antiphospholipase syndrome);

11) Previous pregnancy history and parity;

12) The interval between pregnancies;

13) Systolic blood pressure ≥130 mmHg or diastolic blood pressure ≥80mmHg;

14) The presence of hypertension risk factors such as obstructive sleep apnea;

15) Previous prenatal diagnosis experience (villus biopsy/amniotic fluid puncture).

1.2 Laboratory Examination

Folic acid, vitamin B12, fasting blood glucose, blood routine, urine routine, kidney function, blood lipid, coagulation function, liver function, thyroid function.

1.3 Physical examination

Blood pressure and BMI (height and weight) at 11-13+6 weeks of pregnancy.

1.4 Ultrasonic examination

Uterine artery pulse index, head and hip length, NT at 11-13+6 weeks of pregnancy.

1.5 Serum marker examination

Placental growth factor (PLGF) at 11-13+6 gestational weeks.

1.6 Baseline of primary outcomes

24-hour ambulatory blood pressure, ActiGraph WGT33-BT exercise accelerometer to monitor exercise intensity and energy metabolism.

**Follow-up data registration (middle and late pregnancy)**

2.1 Primary outcomes

24-hour ambulatory blood pressure, ActiGraph WGT33-BT exercise accelerometer to monitor exercise intensity and energy metabolism.

2.2 The presentation of preeclampsia

Systolic blood pressure ≥140mmHg and/or diastolic blood pressure ≥90mmHg after 20 weeks of gestation, accompanied by any of the following: urinary protein quantity ≥0.3g/24h, or urinary protein/creatinine ratio ≥0.3, or urinary protein ≥ (+); or accompanied by following damages of organs involved: heart, lung, liver, kidney and other important organs, or abnormal in the digestive system, nervous system, placenta - fetal involvement, etc.

**Severe pre-eclampsia refers to a pregnant woman with one of the following symptoms:**

1) Uncontrolled blood pressure: systolic blood pressure ≥160mmHg and/or diastolic blood pressure ≥110mmHg;

2) Persistent headache, visual disturbance, or other central nervous system abnormalities;

3) Persistent upper abdominal pain, subcapsular hematoma or liver rupture;

4) Elevated serum alanine aminotransferase (ALT) or aspartate aminotransferase (AST) levels;

5) Impaired renal function: urinary protein content > 2.0g/24h; oliguria (24h urine volume < 400ml, or < 17ml/hour), or serum creatinine level > 106μmol/L;

6) Hypoproteinemia with ascites, pleural effusion or pericardial effusion;

7) Platelet count decreased and lower than 100×10^9^/L; hemolysis, anemia, elevated lactate dehydrogenase (LDH), or jaundice;

8) Heart failure;

9) Pulmonary edema;

10) Fetal growth restriction or oligohydramnios, fetal death, placental abruption, etc.

2.3 Laboratory examination

Fasting blood glucose, blood routine, urine routine, kidney function, coagulation function, liver function, thyroid function.

2.4 Physical examination

Blood pressure, height, weight.

2.5 Ultrasound Examination

Crown-rump length, double parietal diameter, umbilical artery pulse index, umbilical artery resistance index, umbilical artery S/D ratio.

2.6 Screening of serum markers

Serum placental growth factor (PLGF) at 26-27+6 weeks and 32-36+6 weeks.

2.7 Medication compliance

Take the medication prescribed by the doctor at each follow-up visit, and make statistics on the number of drugs taken.

2.8 Other

Abdominal circumference

**Data Management**

An electronic collection and management system is proposed for this study, in which researchers are responsible for the collection of primary data and for organizing the entry of case report information into a spreadsheet. The problems found in the process of data management will be notified to the researchers in the form of a query form, and the researchers will review and give feedback to the questions as soon as possible. The feedback records will be saved as the basis for data modification. After the case review is completed, the data will be locked, and the locked data files are not allowed to be changed.

**Statistical Analysis**

**Data quality assessment and data clearing**

The research requires evaluating whether the integrity, accuracy and consistency of data meet the pre-set quality requirements, and clearing up the duplicate data and unqualified data to form usable data.

**Data analysis**

The study is powered for the primary outcome measure of change in 24-hour ambulatory SBP from the first to the third trimester. The sample size calculation was based on the results of Lopes S et al.[14], who showed a decrease of -7.3 ± 12.7mmHg in 24-hour ambulatory SBP in the exercise group with 1.1 ± 8.2mmHg in the control group. A sample of 72 pregnant women (36 per group), with a two-sided significance level of 0.05, provided 90% statistical power to demonstrate this difference in 24-hour ambulatory SBP. In order to accommodate a 20% attrition rate, 90 pregnant women will be recruited (PASS11, independent t-tests; allocation ratio = 1).

Continuous variables will be presented as means ± standard deviations or median (interquartile range) according to normal distribution or not. Between-group differences at baseline will be tested with Student’s independent t-test or Mann–Whitney U test. For the categorical variables, results will be expressed as counts and percentages. Categorical variables will be tested with the chi-square test or Fischer test if appropriate between groups at baseline. The assessment of compliance with daily physical activity and safety will be summarized for the two groups. A cut-off of 80% of baseline physical activity during the second and third trimesters will be used to determine compliance well. Adverse events, if any, will be reported. The primary outcome and difference of the change in 24-hour ambulatory SBP from the first to the second trimester between groups will be analyzed using analysis of covariance, adjusting for baseline blood pressure. Other secondary outcomes will be analysis using general linear modelling to compare the difference of blood pressure change in the MPA and LPA groups at the second and third trimesters, adjusting for baseline blood pressure. The analyses will be conducted based on the intention-to-treat principle. The intention-to-treat population will comprise all the patients with the exception of those who do not complete ambulatory blood pressure measurement and physical activity monitoring at 3-time points for any reason, such as miscarriage, or preterm birth. Per-protocol set will be performed as a sensitivity analysis. Relative risks and 95% confidence intervals will also be provided. A two-sided p-value below 0.05 will be considered statistically significant in all analyses. Data will be analyzed using SPSS 25 statistical software.

**Study quality control**

During the research process, regular inspection will be carried out to ensure that all contents of the research program are strictly complied with the protocol and that the filling of research data is correct and standardized.

1) Prior to the study, researchers should be trained and the recording methods and judgment standards should be unified. The training content includes but is not limited to research protocols, standard operating procedures involved in the study, study questionnaires and forms, informed consent, ethics, adverse event reports, knowledge of related diseases, and medical knowledge.

2) The researcher shall record all contents in the research form truthfully, and ensure the authenticity and reliability of the contents.

3) All observations in studies should be verified to ensure the reliability of data and ensure that all conclusions in clinical studies have corresponding original records. There are corresponding data management measures in the clinical research and data processing stage.

4) Periodically sample the case report form and check the original documents.

5) In order to ensure the safety of participants and ensure the accuracy, integrity and reliability of data, researchers should keep the original case observation table, clinical medical records and patients' visit records as the original records of this study. Researchers should be able to provide these original records upon request.

**Informed consent, ethical review and legal issues**

1. Informed Consent

The researchers should ensure that the enrolled pregnant women fully understand the nature of the study, the content of the study, the content of the examination, the duration of the study and data ownership, the possible risks and benefits, privacy protection, etc., and obtain written informed consent with the signed name and date. Ensure that patients have the opportunity to ask questions and time to consider the content of informed consent, and ensure that any questions patients may have during the study are answered promptly.

2. Ethical Review

The final study protocol, final written informed consent, and other relevant written information provided to patients must be approved and agreed upon in writing by the Ethics Committee prior to the initiation of studies. The investigator must ensure that these documents are submitted to the appropriate ethics committee and institute staff. The views of the Ethics committee must be expressed in writing.

3. Laws and Regulations

The implementation of this study is in accordance with the Declaration of Helsinki, the Technical Guidelines for Clinical Research on in vitro diagnostic reagents and the ethical principles of relevant laws and regulations on clinical trials of diagnostic reagents in China.

**Data preservation**

In order to ensure the evaluation and supervision of clinical studies by relevant national and responsible units, investigators should agree to keep all research-related data, including patient confirmation, original signed informed consent, detailed records of study forms, etc. The storage period is 3 years after the end of the study.

**Confidentiality and data publication**

The study protocol, investigator's handbook, subject information and any study data generated prior to publication of the study data are confidential and shall not be disclosed. The data generated in this study belong to the research institution and the sponsor, and the research institution has the right to publish the data related to this study. Researchers in the project have the right to sign their names in published research works.

Version number: V2.0; Version date: 2022-07-05

**Daily physical activity on ambulatory blood pressure in pregnant women with chronic hypertension**

**Background**

Pre-eclampsia, which is the second leading cause of maternal death [1], increases the risk of fetal growth restriction, iatrogenic premature delivery, and long-term cardiovascular diseases [2], and threatens the health of the mother and child. The etiology of preeclampsia remains unclear. There is no effective method for expectant treatment except termination of pregnancy which inevitably leads to iatrogenic premature delivery. Therefore, early prediction and prevention are important. Guidelines involving exercise during pregnancy indicate that exercise reduces the incidence of preeclampsia. Therefore, exercise in pregnant women with chronic hypertension may be beneficial to the prevention of preeclampsia and blood pressure control.

Chronic hypertension is estimated to affect 0.3% to 4.3% of pregnant women, and the risk of preeclampsia is 5.43 times in women with chronic hypertension than in those with normotensive. Furthermore, chronic hypertension was associated with a higher risk of cesarean section, maternal mortality, preterm birth, stillbirth, small for gestational age (SGA), low birth weight, and neonatal intensive unit admission [3, 4]. The abovementioned maternal and adverse perinatal outcomes are prone to occur in chronic hypertension-superimposed preeclampsia compared with preeclampsia alone [5, 6]. Blood pressure management is substantial throughout the perinatal period of pregnant women with chronic hypertension. Two large randomized trials [7, 8] found that antihypertensive treatment targeting a blood pressure <140/90 mmHg in chronic hypertension was safe and beneficial for maternal and neonatal. However, some agents should be avoided in pregnancy owing to the possible or proven concerns about fetotoxicity [9-11]. Pregnant women have limited options for antihypertensive medications compared with non-gestational. Pregnant termination should be considered to avoid maternal and fetal complications such as placental abruption and cardiovascular and cerebrovascular adverse events when hypertension is uncontrollable after active treatment. Lifestyle modifications can enhance the effects of antihypertensive therapy. Guidelines for gestational hypertension of pregnancy and non-gestational [12-14] had recommended lifestyle modifications, including salt reduction, smoking and alcohol cessation, healthy diet and drinking, weight reduction, and regular physical activity.

Exercise plays an important role in maintaining and improving cardiopulmonary function and reducing the risk of obesity, diabetes, hypertension and other chronic diseases at all stages of life. Pregnant women are also advised to maintain moderate exercise. Pregnancy exercise reduces joint edema, enhances physical strength during delivery, relieves pain, and reduces the incidence of cesarean section. Exercise during pregnancy also reduces depression. Moderate exercise during pregnancy does not increase premature birth and is helpful to control weight gain during pregnancy, reduce insulin resistance, and prevent gestational diabetes, preeclampsia and other pregnancy complications [15,16]. The consensus on exercise during pregnancy suggests that pregnant women without exercise contraindications should carry out moderate-intensity exercise lasting 30 minutes for 5 days every week [17]. Multiple guidelines have proposed that exercise during pregnancy could reduce the incidence of preeclampsia [17-21]. A meta-analysis showed that exercise reduced the risk of preeclampsia by 41% (OR 0.59, 95% CI 0.37 to 0.94) [15]. For pregnant women diagnosed with preeclampsia, preeclampsia is a contraindication for pregnancy exercise. However, the advantages and disadvantages of pregnancy exercise for such women need to be further confirmed. Further research is also needed to determine whether exercise interventions benefit blood pressure control in women with preeclampsia.

Globally, exercise is recommended as a first-line adjunct to the control of non-gestational hypertension [22-23]. A large number of studies have confirmed that exercise intervention can reduce blood pressure in adults with prehypertension and hypertension, and also reduce the risk of hypertension in adults with normal blood pressure. A meta-analysis [24] showed that exercise intervention can significantly reduce both systolic and diastolic blood pressure in prehypertension, and reduce systolic blood pressure by 2-5mmHg and diastolic blood pressure by 1-4mmHg in adults with normal blood pressure. Studies [25] have also shown that high-intensity daily physical activity is associated with a lower incidence of hypertension. A recent RCT study [26] published in JAMA Cardiology showed that 12 weeks of moderate-intensity aerobic exercise was effective in reducing blood pressure in patients with refractory hypertension. At the same time, more and more evidence has shown that reduced systolic blood pressure in adults with hypertension is linearly correlated with the reduction of cardiovascular disease morbidity and mortality [27]. A reduction of 10mmHg in systolic blood pressure or 4mmHg in diastolic blood pressure can reduce the risk of stroke by about 30% and myocardial infarction by 20% [28]. Therefore, exercise plays an important role in the prevention and control of hypertension and its cardiovascular and cerebrovascular complications.

The management of blood pressure is an important part of the diagnosis and treatment of pregnant women with chronic hypertension. Due to the potential impact on the fetus, the options for pregnant women with chronic hypertension are limited. Termination of pregnancy should be considered when hypertension is uncontrollable to avoid serious maternal and infant complications such as cardiovascular and cerebrovascular accidents and placental abruption. Exercise as a first-line adjunct to the management of non-pregnant hypertension may benefit pregnant women with chronic hypertension. Previous studies [29] have found that exercise can benefit the blood pressure of pregnant women with normal blood pressure in late pregnancy. An observational cohort study suggests that adherence to a beneficial lifestyle may significantly reduce the risk of chronic hypertension after gestational hypertension disease (HDP), but there is no clear evidence that physical exercise can alter the relationship between HDP and chronic hypertension. Two studies [30,31] of 116 pregnant women with chronic hypertension and/or pre-eclampsia showed that once-a-week physical exercise using a stationary bicycle had no effect on changes in delivery mode, maternal and neonatal morbidity, blood pressure and heart rate. There are few studies available to guide pregnancy care for women with chronic hypertension. Research is needed to explore and help address questions about physical activity during pregnancy in women with chronic hypertension, such as benefits and harms, how often, how far, for how long, and at what intensity.

This study aims to explore the influence of physical activity on blood pressure of pregnant women with chronic hypertension by monitoring the change of daily physical activity intensity and blood pressure during pregnancy, and further provide evidence for exercise during pregnancy of pregnant women with chronic hypertension.

**Reference**

1. Say L, Chou D, Gemmill A, et al. Global causes of maternal death: a WHO systematic analysis. Lancet Glob Health, 2014, 2(6): e323-33.
2. Phipps EA, Thadhani R, Benzing T, et al. Pre-eclampsia: Pathogenesis, novel diagnostics and therapies. Nat Rev Nephrol, 2019, 15(5): 275-28.
3. Al Khalaf SY, O'Reilly EJ, Barrett PM, DF BL, Pawley LC, McCarthy FP, et al. Impact of Chronic Hypertension and Antihypertensive Treatment on Adverse Perinatal Outcomes: Systematic Review and Meta-Analysis. J Am Heart Assoc. 2021;10(9):e018494.
4. Bramham K, Parnell B, Nelson-Piercy C, Seed PT, Poston L, Chappell LC. Chronic hypertension and pregnancy outcomes: systematic review and meta-analysis. BMJ. 2014;348:g2301.
5. Valent AM, DeFranco EA, Allison A, Salem A, Klarquist L, Gonzales K, et al. Expectant management of mild preeclampsia versus superimposed preeclampsia up to 37 weeks. Am J Obstet Gynecol. 2015;212(4):515 e1-8.
6. Rezk M, Gamal A, Emara M. Maternal and fetal outcome in de novo preeclampsia in comparison to superimposed preeclampsia: a two-year observational study. Hypertens Pregnancy. 2015;34(2):137-44.
7. Magee LA, von Dadelszen P, Rey E, Ross S, Asztalos E, Murphy KE, et al. Less-tight versus tight control of hypertension in pregnancy. N Engl J Med. 2015;372(5):407-17.
8. Tita AT, Szychowski JM, Boggess K, Dugoff L, Sibai B, Lawrence K, et al. Treatment for Mild Chronic Hypertension during Pregnancy. N Engl J Med. 2022;386(19):1781-92.
9. Bellos I, Pergialiotis V, Papapanagiotou A, Loutradis D, Daskalakis G. Comparative efficacy and safety of oral antihypertensive agents in pregnant women with chronic hypertension: a network metaanalysis. Am J Obstet Gynecol. 2020;223(4):525-37.
10. Ahmed B, Tran DT, Zoega H, Kennedy SE, Jorm LR, Havard A. Maternal and perinatal outcomes associated with the use of renin-angiotensin system (RAS) blockers for chronic hypertension in early pregnancy. Pregnancy Hypertens. 2018;14:156-61.
11. Bateman BT, Patorno E, Desai RJ, Seely EW, Mogun H, Dejene SZ, et al. Angiotensin-Converting Enzyme Inhibitors and the Risk of Congenital Malformations. Obstet Gynecol. 2017;129(1):174-84.
12. Hypertensive Disorders in Pregnancy Subgroup CSoO, Gynecology CMA. [Diagnosis and treatment of hypertension and pre-eclampsia in pregnancy: a clinical practice guideline in China2020]. Zhonghua Fu Chan Ke Za Zhi. 2020;55(4):227-38.
13. ACOG Practice Bulletin No. 202: Gestational Hypertension and Preeclampsia. Obstet Gynecol. 2019;133(1):1.
14. Unger T, Borghi C, Charchar F, Khan NA, Poulter NR, Prabhakaran D, et al. 2020 International Society of Hypertension Global Hypertension Practice Guidelines. Hypertension. 2020;75(6):1334-57.
15. Davenport MH, Ruchat SM, Poitras VJ, et al. Prenatal exercise for the prevention of gestational diabetes mellitus and hypertensive disorders of pregnancy: a systematic review and meta-analysis[J]. Br J Sports Med, 2018,52(21):1367-1375.
16. Wang C, Wei Y, Zhang X, et al. A randomized clinical trial of exercise during pregnancy to prevent gestational diabetes mellitus and improve pregnancy outcome in overweight and obese pregnant women[J]. Am J Obstet Gynecol, 2017, 216(4):340-351.
17. 中国妇幼保健协会妊娠合并糖尿病专业委员会,中华医学会妇产科学分会产科学组. 妊娠期运动专家共识(草案). 中华围产医学杂志,2021,24(09):641-645.
18. ACOG Committee Opinion No. 650: Physical activity and exercise during pregnancy and the postpartum period[J]. Obstet Gynecol, 2015,126(6):e135-142.
19. Mottola MF, Davenport MH, Ruchat SM, et al. 2019 Canadian guideline for physical activity throughout pregnancy[J]. Br J Sports Med, 2018, 52(21): 1339-1346.
20. Royal College of Obstetricians and Gynaecologists. Exercise in pregnancy (Statement No. 4) [EB/OL]. (2015-02-04) [2021-06-01]. https:// www. rcog. org. uk/en/guidelines-research-services/guidelines/exercise-in-pregnancy-statement-no.4/.
21. Evenson KR, Barakat R, Brown WJ, et al. Guidelines for physical activity during pregnancy: comparisons from around the world[J]. Am J Lifestyle Med, 2014,8(2):102-121.
22. Williams B, Mancia G, SpieringW, et al; ESC Scientific Document Group. 2018 ESC/ESH guidelines for the management of arterial hypertension. Eur Heart J. 2018;39(33):3021-3104.
23. Whelton PK, Carey RM, Aronow WS, et al. 2017 ACC/AHA/AAPA/ABC/ACPM/AGS/APhA/ASH/ASPC/NMA/PCNA guideline for the prevention, detection, evaluation, and management of high blood pressure in adults: a report of the American College of Cardiology/American Heart Association Task Force on Clinical Practice Guidelines. Circulation.2018;138(17): e484-e594.
24. Pescatello, LS; Buchner, DM; Jakicic, JM; et al. Physical Activity to Prevent and Treat Hypertension: A Systematic Review. Med Sci Sports Exerc.2019 06 ;51(6) :1314-1323.
25. Huai P, Xun H, Reilly KH, Wang Y, Ma W, Xi B. Physical activity and risk of hypertension: a meta-analysis of prospective cohort studies.Hypertension. 2013;62(6):1021–6.
26. Lopes, S; Mesquita-Bastos, J; Garcia, C; et al. Effect of Exercise Training on Ambulatory Blood Pressure Among Patients with Resistant Hypertension A Randomized Clinical Trial. JAMA Cardiol.2021 11 01 ;6(11) :1317-1323.
27. Bundy JD, Li C, Stuchlik P, et al. Systolic blood pressure reduction and risk of cardiovascular disease and mortality: a systematic review and network meta-analysis. JAMA Cardiol. 2017;2(7):775-781.
28. Staessen JA, Wang JG, Thijs L. Cardiovascular protection and blood pressure reduction: ameta-analysis. Lancet. 2001;358(9290):1305-1315.
29. Sobierajski, FM; Purdy, GM; Usselman, CW; et al. Maternal Physical Activity Is Associated with Improved Blood Pressure Regulation During Late Pregnancy. Can J Cardiol.2018 04 ;34(4) :485-491
30. Kasawara KT, Burgos CS, do Nascimento SL, Ferreira NO, Surita FG, Pinto ESJL. Maternal and Perinatal Outcomes of Exercise in Pregnant Women with Chronic Hypertension and/or Previous Preeclampsia: A Randomized Controlled Trial. ISRN Obstet Gynecol. 2013;2013:857047.
31. Burgos CS, Kasawara KT, Costa ML, Pinto ESJL. PP041. The effect of exercise in pregnant women with chronic hypertension and/or previous preeclampsia on blood pressure and heart rate variability. Pregnancy Hypertens. 2012;2(3):263-4.

**Method**

**Inclusion criteria**

Maternal age ≥ 18 years old, singleton pregnancy and fetal survival at 11^+0^ to 13^+6^ weeks of gestational age, history of chronic hypertension defined as hypertension diagnosed before pregnancy or at the first prenatal visit.

**Exclusion criteria**

Severe cardiovascular or respiratory disease, hyperthyroidism, pregestational diabetes mellitus, incompetent cervix, recurrent spontaneous abortion, a history of spontaneous preterm birth, threatened abortion or inevitable abortion, placenta praevia, severe anemia, malnutrition or very low body weight (body mass index < 12kg/m2), with severe mental disorders and disable to express their will, with other obvious abnormal signs, laboratory examination and other clinical diseases.

**Diagnostic criteria** (2020 Chinese Guidelines for the Diagnosis and Treatment of Hypertensive Diseases during Pregnancy)

**Preeclampsia**

Systolic blood pressure ≥140mmHg and/or diastolic blood pressure ≥90mmHg after 20 weeks of gestation, accompanied by any of the following: urinary protein quantity ≥0.3g/24h, or urinary protein/creatinine ratio ≥0.3, or urinary protein ≥ (+); or accompanied by following damages of organs involved: heart, lung, liver, kidney and other important organs, or abnormal in the digestive system, nervous system, placenta - fetal involvement, etc.

**Severe pre-eclampsia refers to a pregnant woman with one of the following symptoms:**

1) Uncontrolled blood pressure: systolic blood pressure ≥160mmHg and/or diastolic blood pressure ≥110mmHg;

2) Persistent headache, visual disturbance, or other central nervous system abnormalities;

3) Persistent upper abdominal pain, subcapsular hematoma or liver rupture;

4) Elevated serum alanine aminotransferase (ALT) or aspartate aminotransferase (AST) levels;

5) Impaired renal function: urinary protein content > 2.0g/24h; oliguria (24h urine volume < 400ml, or < 17ml/hour), or serum creatinine level > 106μmol/L;

6) Hypoproteinemia with ascites, pleural effusion or pericardial effusion;

7) Platelet count decreased and lower than 100×10^9^/L; hemolysis, anemia, elevated lactate dehydrogenase (LDH), or jaundice;

8) Heart failure;

9) Pulmonary edema;

10) Fetal growth restriction or oligohydramnios, fetal death, placental abruption, etc.

**End events**

1) Termination of pregnancy

2) The following conditions occur: vaginal bleeding, regular and painful contractions, premature rupture of membranes, dyspnea, dizziness, headache, chest pain, muscle weakness affecting balance

3) Other serious clinical diseases which are not suitable for further study.

**Grouping scheme**

1. Grouping method: During early pregnancy (11~13+6 weeks), an ActiGraph wGT3X-BT exercise accelerometer will be worn on the wrist to monitor the intensity, time, number of steps and energy metabolism of daily physical activity within 1 week, and the number of exercise steps will be collected by mobile phone APP. Participants will be divided into light and moderate groups according to the activity intensity of early pregnancy. Moderate-intensity activity is defined as 150 minutes per week, and the light-intensity activity group has a activity intensity or activity time of fewer than 150 minutes per week.

2. Drug intervention plan: pregnant women in both groups take low-dose aspirin 100mg daily from 11 to 13+6 gestational weeks until 2 to 7 days before termination or 34 gestational weeks as recommended by the guidelines. Oral calcium supplementation should be at least 1 g/ day.

3. Health guidance: limit salt intake (<6 g/d), smoking cessation, regular rest, weight control, etc.

4. Exercise monitoring: Wear an ActiGraph wGT3X-BT exercise accelerometer on wrist to monitor physical activity for a week. Except for bathing, swimming and other contacts with water, the rest of the time should be worn as required, ensure that more than 10 hours of wear every day, and at least two working days (Monday to Friday), rest day (Saturday to Sunday) with one day of valid data.

1) Activity intensity: light physical activity (100 -1952 CPM) and moderate physical activity (≥1952 CPM). Data were collected by ActiLife 6.0 analysis software after completion.

2) Active metabolic rate: measured by average metabolic equivalents (METs).

3) Perceived exertion rating: ratings of perceived exertion (RPE) based on the Borg perceived Exertion Scale (Table 1) are used. The Borg scale has 15 ratings ranging from 6 to 20, representing different levels of fatigue, with 6 being "very, very easy" and 20 beings "very, very difficult". For moderate-intensity exercise, the RPE score of pregnant women should be 13 to 14 points, that is, the feeling of self-exercise intensity is somewhat difficult.


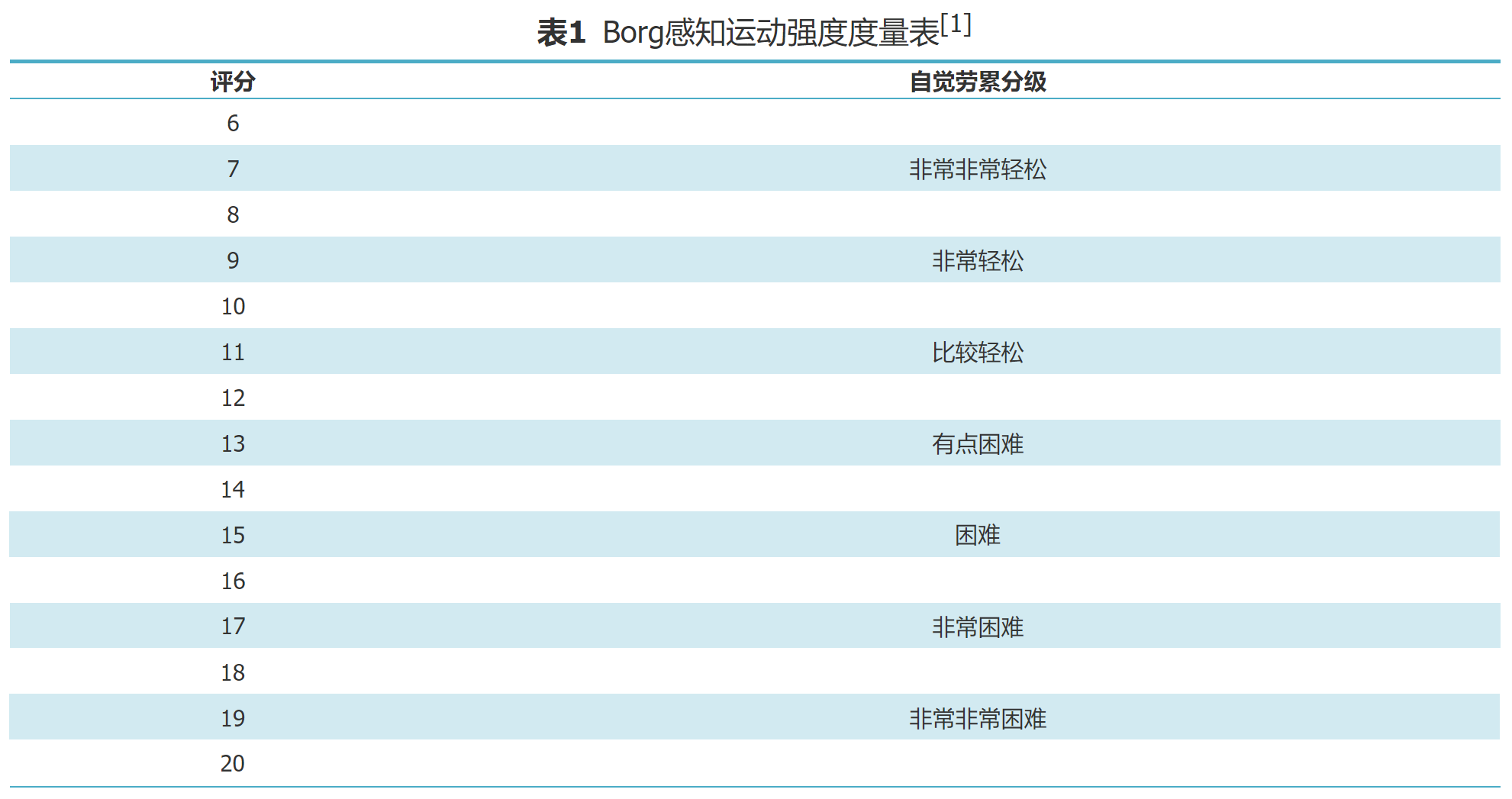


**Outcomes**

1. Primary outcome: the difference in 24-hour ambulatory systolic blood pressure change from baseline during the third trimesters of pregnancy;

2. Secondary outcomes: the difference in 24-hour ambulatory systolic blood pressure change from baseline during the second trimesters of pregnancy; the difference of daytime and nighttime ambulatory blood pressure, daytime and nighttime and 24-hour diastolic blood pressure, and clinic blood pressure changes from baseline;

3. Exploratory outcomes: incidence of preeclampsia; effect of exercise on blood pressure control in pregnant women with preeclampsia

4. Safety outcomes: activity tolerance decline rate; incidence of shortness of breath; incidence of heart failure;

5. Other relevant outcomes: severe complications of preeclampsia (eclampsia, heart failure, pulmonary edema, HELLP syndrome, DIC), fetal death, iatrogenic premature delivery, neonatal asphyxia, NICU. Compliance with oral aspirin and calcium; mobile phone exercise recording monitoring exercise intensity compliance; the Subjective Exertion Scale (RPE) monitors an individual's exercise intensity tolerance.


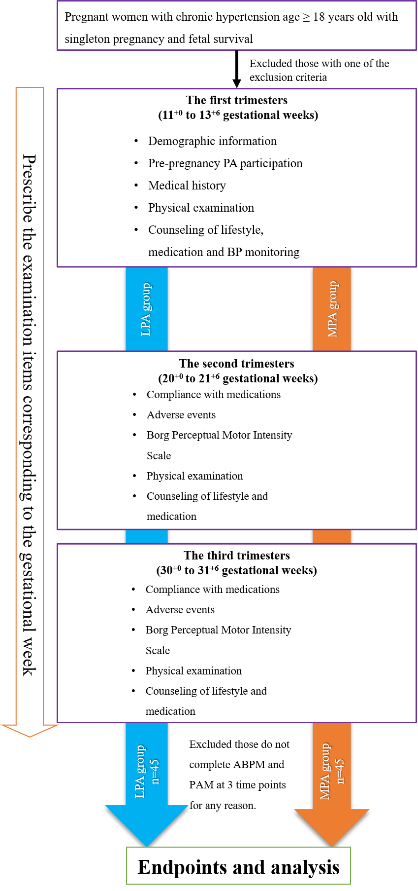
**Procedure**

**Time and content of information collection**

1 Registration Information (11-13+6)

1.1 Demographic characteristics and medical history of pregnant women (risk factors)

1) Age, race, height, weight, gestational age (CRL calculation method), occupation;

2) Method of pregnancy (natural pregnancy, assisted reproductive technology - using ovulation drugs or in vitro fertilization);

3) Smoking history;

4) History of chronic hypertension;

5) History of type I or type II diabetes;

6) History of kidney disease;

7) Abnormal pregnancy history (21/18-trisomy, NTD, unknown abortion history, gestational diabetes, etc.);

8) History of preeclampsia;

9) Family history of preeclampsia (mother or sister);

10) History of autoimmune disease (systemic lupus erythematosus or antiphospholipase syndrome);

11) Previous pregnancy history and parity;

12) The interval between pregnancies;

13) Systolic blood pressure ≥130 mmHg or diastolic blood pressure ≥80mmHg;

14) The presence of hypertension risk factors such as obstructive sleep apnea;

15) Previous prenatal diagnosis experience (villus biopsy/amniotic fluid puncture).

1.2 Laboratory Examination

Folic acid, vitamin B12, fasting blood glucose, blood routine, urine routine, kidney function, blood lipid, coagulation function, liver function, thyroid function.

1.3 Physical examination

Blood pressure and BMI (height and weight) at 11-13+6 weeks of pregnancy.

1.4 Ultrasonic examination

Uterine artery pulse index, head and hip length, NT at 11-13+6 weeks of pregnancy.

1.5 Serum marker examination

Placental growth factor (PLGF) at 11-13+6 gestational weeks.

1.6 Baseline of primary outcomes

24-hour ambulatory blood pressure, ActiGraph WGT33-BT exercise accelerometer to monitor exercise intensity and energy metabolism.

**Follow-up data registration (middle and late pregnancy)**

2.1 Primary outcomes

24-hour ambulatory blood pressure, ActiGraph WGT33-BT exercise accelerometer to monitor exercise intensity and energy metabolism.

2.2 The presentation of preeclampsia

Systolic blood pressure ≥140mmHg and/or diastolic blood pressure ≥90mmHg after 20 weeks of gestation, accompanied by any of the following: urinary protein quantity ≥0.3g/24h, or urinary protein/creatinine ratio ≥0.3, or urinary protein ≥ (+); or accompanied by following damages of organs involved: heart, lung, liver, kidney and other important organs, or abnormal in the digestive system, nervous system, placenta - fetal involvement, etc.

**Severe pre-eclampsia refers to a pregnant woman with one of the following symptoms:**

1) Uncontrolled blood pressure: systolic blood pressure ≥160mmHg and/or diastolic blood pressure ≥110mmHg;

2) Persistent headache, visual disturbance, or other central nervous system abnormalities;

3) Persistent upper abdominal pain, subcapsular hematoma or liver rupture;

4) Elevated serum alanine aminotransferase (ALT) or aspartate aminotransferase (AST) levels;

5) Impaired renal function: urinary protein content > 2.0g/24h; oliguria (24h urine volume < 400ml, or < 17ml/hour), or serum creatinine level > 106μmol/L;

6) Hypoproteinemia with ascites, pleural effusion or pericardial effusion;

7) Platelet count decreased and lower than 100×10^9^/L; hemolysis, anemia, elevated lactate dehydrogenase (LDH), or jaundice;

8) Heart failure;

9) Pulmonary edema;

10) Fetal growth restriction or oligohydramnios, fetal death, placental abruption, etc.

2.3 Laboratory examination

Fasting blood glucose, blood routine, urine routine, kidney function, coagulation function, liver function, thyroid function.

2.4 Physical examination

Blood pressure, height, weight.

2.5 Ultrasound Examination

Crown-rump length, double parietal diameter, umbilical artery pulse index, umbilical artery resistance index, umbilical artery S/D ratio.

2.6 Screening of serum markers

Serum placental growth factor (PLGF) at 26-27+6 weeks and 32-36+6 weeks.

2.7 Medication compliance

Take the medication prescribed by the doctor at each follow-up visit, and make statistics on the number of drugs taken.

2.8 Other

Abdominal circumference

**Data Management**

An electronic collection and management system is proposed for this study, in which researchers are responsible for the collection of primary data and for organizing the entry of case report information into a spreadsheet. The problems found in the process of data management will be notified to the researchers in the form of a query form, and the researchers will review and give feedback to the questions as soon as possible. The feedback records will be saved as the basis for data modification. After the case review is completed, the data will be locked, and the locked data files are not allowed to be changed.

**Statistical Analysis**

**Data quality assessment and data clearing**

The research requires evaluating whether the integrity, accuracy and consistency of data meet the pre-set quality requirements, and clearing up the duplicate data and unqualified data to form usable data.

**Data analysis**

The study is powered for the primary outcome measure of change in 24-hour ambulatory SBP from the first to the third trimester. The sample size calculation was based on the results of Lopes S et al.[14], who showed a decrease of -7.3 ± 12.7mmHg in 24-hour ambulatory SBP in the exercise group with 1.1 ± 8.2mmHg in the control group. A sample of 72 pregnant women (36 per group), with a two-sided significance level of 0.05, provided 90% statistical power to demonstrate this difference in 24-hour ambulatory SBP. In order to accommodate a 20% attrition rate, 90 pregnant women will be recruited (PASS11, independent t-tests; allocation ratio = 1).

Continuous variables will be presented as means ± standard deviations or median (interquartile range) according to normal distribution or not. Between-group differences at baseline will be tested with Student’s independent t-test or Mann–Whitney U test. For the categorical variables, results will be expressed as counts and percentages. Categorical variables will be tested with the chi-square test or Fischer test if appropriate between groups at baseline. The assessment of compliance with daily physical activity and safety will be summarized for the two groups. A cut-off of 80% of baseline physical activity during the second and third trimesters will be used to determine compliance well. Adverse events, if any, will be reported. The primary outcome and difference of the change in 24-hour ambulatory SBP from the first to the second trimester between groups will be analyzed using analysis of covariance, adjusting for baseline blood pressure. Other secondary outcomes will be analysis using general linear modelling to compare the difference of blood pressure change in the MPA and LPA groups at the second and third trimesters, adjusting for baseline blood pressure. The analyses will be conducted based on the intention-to-treat principle. The intention-to-treat population will comprise all the patients with the exception of those who do not complete ambulatory blood pressure measurement and physical activity monitoring at 3-time points for any reason, such as miscarriage, or preterm birth. Per-protocol set will be performed as a sensitivity analysis. Relative risks and 95% confidence intervals will also be provided. A two-sided p-value below 0.05 will be considered statistically significant in all analyses. Data will be analyzed using SPSS 25 statistical software.

**Study quality control**

During the research process, regular inspection will be carried out to ensure that all contents of the research program are strictly complied with the protocol and that the filling of research data is correct and standardized.

1) Prior to the study, researchers should be trained and the recording methods and judgment standards should be unified. The training content includes but is not limited to research protocols, standard operating procedures involved in the study, study questionnaires and forms, informed consent, ethics, adverse event reports, knowledge of related diseases, and medical knowledge.

2) The researcher shall record all contents in the research form truthfully, and ensure the authenticity and reliability of the contents.

3) All observations in studies should be verified to ensure the reliability of data and ensure that all conclusions in clinical studies have corresponding original records. There are corresponding data management measures in the clinical research and data processing stage.

4) Periodically sample the case report form and check the original documents.

5) In order to ensure the safety of participants and ensure the accuracy, integrity and reliability of data, researchers should keep the original case observation table, clinical medical records and patients' visit records as the original records of this study. Researchers should be able to provide these original records upon request.

**Informed consent, ethical review and legal issues**

1. Informed Consent

The researchers should ensure that the enrolled pregnant women fully understand the nature of the study, the content of the study, the content of the examination, the duration of the study and data ownership, the possible risks and benefits, privacy protection, etc., and obtain written informed consent with the signed name and date. Ensure that patients have the opportunity to ask questions and time to consider the content of informed consent, and ensure that any questions patients may have during the study are answered promptly.

2. Ethical Review

The final study protocol, final written informed consent, and other relevant written information provided to patients must be approved and agreed upon in writing by the Ethics Committee prior to the initiation of studies. The investigator must ensure that these documents are submitted to the appropriate ethics committee and institute staff. The views of the Ethics committee must be expressed in writing.

3. Laws and Regulations

The implementation of this study is in accordance with the Declaration of Helsinki, the Technical Guidelines for Clinical Research on in vitro diagnostic reagents and the ethical principles of relevant laws and regulations on clinical trials of diagnostic reagents in China.

**Data preservation**

In order to ensure the evaluation and supervision of clinical studies by relevant national and responsible units, investigators should agree to keep all research-related data, including patient confirmation, original signed informed consent, detailed records of study forms, etc. The storage period is 3 years after the end of the study.

**Confidentiality and data publication**

The study protocol, investigator's handbook, subject information and any study data generated prior to publication of the study data are confidential and shall not be disclosed. The data generated in this study belong to the research institution and the sponsor, and the research institution has the right to publish the data related to this study. Researchers in the project have the right to sign their names in published research works.blished research works.
